# Preclinical evaluation of a safety-aware multimodal robotic pipeline for human–robot interaction in post-stroke rehabilitation

**DOI:** 10.3389/fdgth.2026.1951309

**Published:** 2026-10-09

**Authors:** Bruna Maria Vittoria Guerra, Roberto Soldi, Francesco Lunghi, Stefania Sozzi, Leo Russo, Micaela Schmid, Stefano Ramat

**Affiliations:** Bioengineering Laboratory, Department of Electrical, Computer and Biomedical Engineering, University of Pavia, Pavia, Italy

**Keywords:** attentional monitoring, finite-state machine, hemispatial inattention, human–robot interaction, multimodal interaction, post-stroke rehabilitation, rehabilitation robotics

## Abstract

**Introduction:**

Post-stroke rehabilitation increasingly requires technologies capable of supporting intensive, measurable, and adaptable interventions while preserving clinical supervision and patient safety. This study presents a lightweight, safety-aware, and modular multimodal robotic pipeline for embodied human–robot interaction in post-stroke rehabilitation, with a first target application in hemispatial inattention.

**Methods:**

The system was implemented on the TIAGo mobile manipulator within a ROS 2-based architecture and integrates therapist-mediated supervision, speech-based and graphical interaction, head-orientation monitoring, adaptive auditory recall, robot-mediated and screen-based task delivery, and structured performance logging. Dialogue management is based on a deterministic finite-state machine coupled with a rule-based Natural Language Understanding pipeline, enabling predictable interaction handling, explicit fallback behavior, and therapist escalation for safety-relevant events. A preliminary preclinical evaluation was conducted in 16 healthy participants, with a repeated acquisition after 1 month. The study assessed operational feasibility, multimodal interaction robustness, head-orientation monitoring stability, task execution, ASR-related interaction events, usability, acceptance, and 1-month reproducibility of workflow execution.

**Results:**

All participants completed both acquisitions without adverse events or safety-relevant events requiring interruption of the experimental workflow. End-effector recognition accuracy remained high, attention-recall events were infrequent, screen-based visual tasks were successfully administered and logged, and usability and acceptance outcomes were favorable, with low anxiety toward the robotic platform.

**Discussion:**

These findings support the feasibility of using a therapist-supervised robotic platform as an integrated tool for structured interaction, head-orientation monitoring, rehabilitation-oriented task delivery, and quantitative logging. The study presents a preclinical evaluation of system architecture, safety-aware interaction design, and workflow robustness. Future work is organized in two stages: a dedicated acceptance and robustness protocol in healthy controls to formally test interaction acceptance, speech-recognition robustness, and safety-relevant failure handling, followed by a true rehabilitation protocol in post-stroke patients with hemispatial inattention, including therapist-supervised exercise adaptation.

## 1 Introduction

Post-stroke rehabilitation is a complex and highly individualized process involving motor, sensory, cognitive, perceptual, communicative, emotional, and functional domains. Effective rehabilitation therefore requires not only repeated exercise delivery, but also continuous assessment, patient-specific adaptation, monitoring of engagement and fatigue, and close clinical supervision, particularly in the early and subacute phases after stroke, when patients may be medically fragile, cognitively fatigued, bedridden, or wheelchair-bound (1, 2).

Robotic and intelligent technologies have increasingly been proposed to support both objective assessment and personalized intervention in neurorehabilitation. Rehabilitation robots can deliver structured, repeatable, and measurable activities while collecting quantitative information related to movement quality, response timing, task completion, user engagement, and performance (3–5). However, the clinical impact of robot-assisted therapy remains nuanced. Recent reviews suggest that improvements in impairment-level outcomes do not always translate into comparable gains in activities of daily living, while non-motor dimensions, including cognition, motivation, anxiety, and self-efficacy, are increasingly recognized as factors impacting quality of life in robotic rehabilitation (6–8). These findings indicate that rehabilitation robotics should move beyond repetitive motor assistance alone and integrate interaction, motivation, cognitive engagement, context-aware monitoring, and therapist supervision.

Within this perspective, Human–Robot Interaction (HRI) becomes a core component of rehabilitation robotics rather than a secondary interface layer. A robot used in a clinical rehabilitation setting should explain the session, request explicit confirmation, guide the patient through the task, provide corrective or motivational feedback, manage misunderstandings, and react appropriately to fatigue, refusal, confusion, malaise, or requests for help. Socially Assistive Robotics (SAR) addresses this need by introducing robots that support users through social, cognitive, and motivational interaction rather than through physical assistance alone (9, 10). In post-stroke rehabilitation, robot-mediated interaction has been used to support repeated training, feedback, and patient motivation (11, 12). Although recent studies on Large Language Models (LLMs) in socially assistive robots have highlighted their potential to increase conversational flexibility and user-perceived acceptability, their use in safety-relevant rehabilitation scenarios also raises additional requirements related to latency, transparency, supervision, and controllability (13, 14).

Among post-stroke cognitive and perceptual disorders, hemispatial inattention, also referred to as unilateral spatial neglect, represents a particularly relevant target for interaction-driven robotic rehabilitation. Patients with hemispatial inattention may show reduced spontaneous exploration of the contralesional hemispace, impaired visual scanning, diminished responsiveness to external cues, and reduced awareness of their deficits. These impairments affect activities of daily living, mobility, environmental exploration, and participation in rehabilitation. From an HRI perspective, this condition is especially meaningful because attentional engagement, spatial cueing, visual exploration, verbal recall, and therapist-guided supervision are central components of the rehabilitation process (15, 16).

Recent reviews on spatially structured technologies for unilateral spatial neglect have emphasized the potential of virtual reality, augmented reality, and artificial intelligence to provide more ecological rehabilitation scenarios than conventional paper-and-pencil or flat-screen tasks (5, 15, 16). In this context, an embodied robotic platform may further extend spatially structured rehabilitation: the robot's physical position, voice, screen, arm, and end-effector can act as coordinated attentional cues, encouraging the patient to orient toward the target hemispace during verbal interaction, visual exploration, or object-based tasks.

Despite these opportunities, the interaction architecture required to coordinate a real robotic rehabilitation workflow remains challenging. Recent progress in Natural Language Processing (NLP), Natural Language Understanding (NLU), LLMs, and language-conditioned robotics has opened new opportunities for flexible human–robot communication and semantic interpretation (14, 17, 18). In embodied rehabilitation robotics, however, dialogue management has to be integrated with navigation, RGB-D perception, face tracking, speech recognition, speech synthesis, graphical interface management, exercise execution, logging, and communication with therapist interfaces. These concurrent processes require interaction architectures that can operate with bounded computational cost, transparent decision logic, and stable behavior during safety-relevant interaction phases (19).

In this context, conversational HRI for rehabilitation can benefit from constrained and hybrid architectures, where language interpretation supports interaction flexibility while an explicit dialogue policy maintains traceability, therapist supervision, and controlled recovery from ambiguous inputs (20, 21). This is particularly relevant when patients express fatigue, confusion, distress, refusal, or discomfort through short, fragmented, or semantically ambiguous utterances. The interaction architecture must therefore support not only task progression, but also confirmation, clarification, fallback handling, and escalation to the therapist when clinically relevant events occur (14, 21).

Building on the broader Fit4MedRob platform for intelligent robotic rehabilitation (22, 23), this paper presents a ROS 2-based multimodal robotic pipeline implemented on the TIAGo mobile manipulator (PAL Robotics, Barcelona, Spain) and designed for therapist-supervised embodied HRI in post-stroke rehabilitation, with a first target application in hemispatial inattention. The present study focuses on the interaction and supervision layer required to operate a rehabilitation-oriented robotic workflow on a real platform. The system is organized around three functional components: a Therapist Control Interface (TCI), an Interaction Module (IM), and an Exercise Delivery Module (EDM), integrated with a head-orientation monitoring subsystem. The contribution of the work lies in the coordinated integration of therapist-mediated supervision, lightweight NLU, FSM-based dialogue and safety management, multimodal synchronization, adaptive attention recall, robot-mediated and screen-based task delivery, structured logging, and therapist escalation for clinically relevant events within a single operational robotic pipeline.

A preliminary evaluation study involving 16 healthy participants was conducted to assess technical feasibility, multimodal interaction robustness, operational integration, usability, acceptance, and 1-month reproducibility of workflow execution before clinical experimentation with patients. Usability and acceptance were evaluated through the System Usability Scale (SUS), the Usability Metric for User Experience-Lite (UMUX-Lite), and selected constructs from the Unified Theory of Acceptance and Use of Technology (UTAUT) (24–27). Interaction robustness was assessed through structured session logs, head-orientation metrics, attentional recall events, end-effector recognition accuracy, screen-based task outcomes, ASR-related interaction-event analysis, and 1-month workflow re-execution comparisons. Overall, the study evaluates the feasibility, usability, and operational robustness of a therapist-supervised multimodal robotic workflow implemented on a real mobile manipulator.

## 2 Materials and methods

### 2.1 Robotic platform and system overview

The rehabilitation framework was implemented on the PAL Robotics TIAGo mobile manipulator within a modular ROS 2-based architecture. The software implementation was developed in the PAL/ROS 2 environment for TIAGo, using ROS 2 Humble as middleware. The robotic platform is equipped with a bidirectional mobile base, LiDAR sensors, an RGB-D camera, onboard microphones, a 7-DOF robotic arm with gripper and an integrated touchscreen mounted on the torso (Figure 1). Onboard computation is provided by an Intel Core i7 processor, 16 GB of RAM, and a 512 GB SSD. These computational resources are shared across several concurrent processes, including autonomous navigation, RGB-D perception, speech synthesis and recognition, graphical interaction, exercise delivery, data logging, and therapist-interface communication. Given such a concurrent onboard workload, the interaction layer was designed as a lightweight and deterministic architecture. The FSM-based dialogue policy was used as the primary control layer for safety-relevant transitions, supporting explicit state progression, bounded decision logic, and stable execution within the robot's onboard computational constraints.

**Figure 1:**
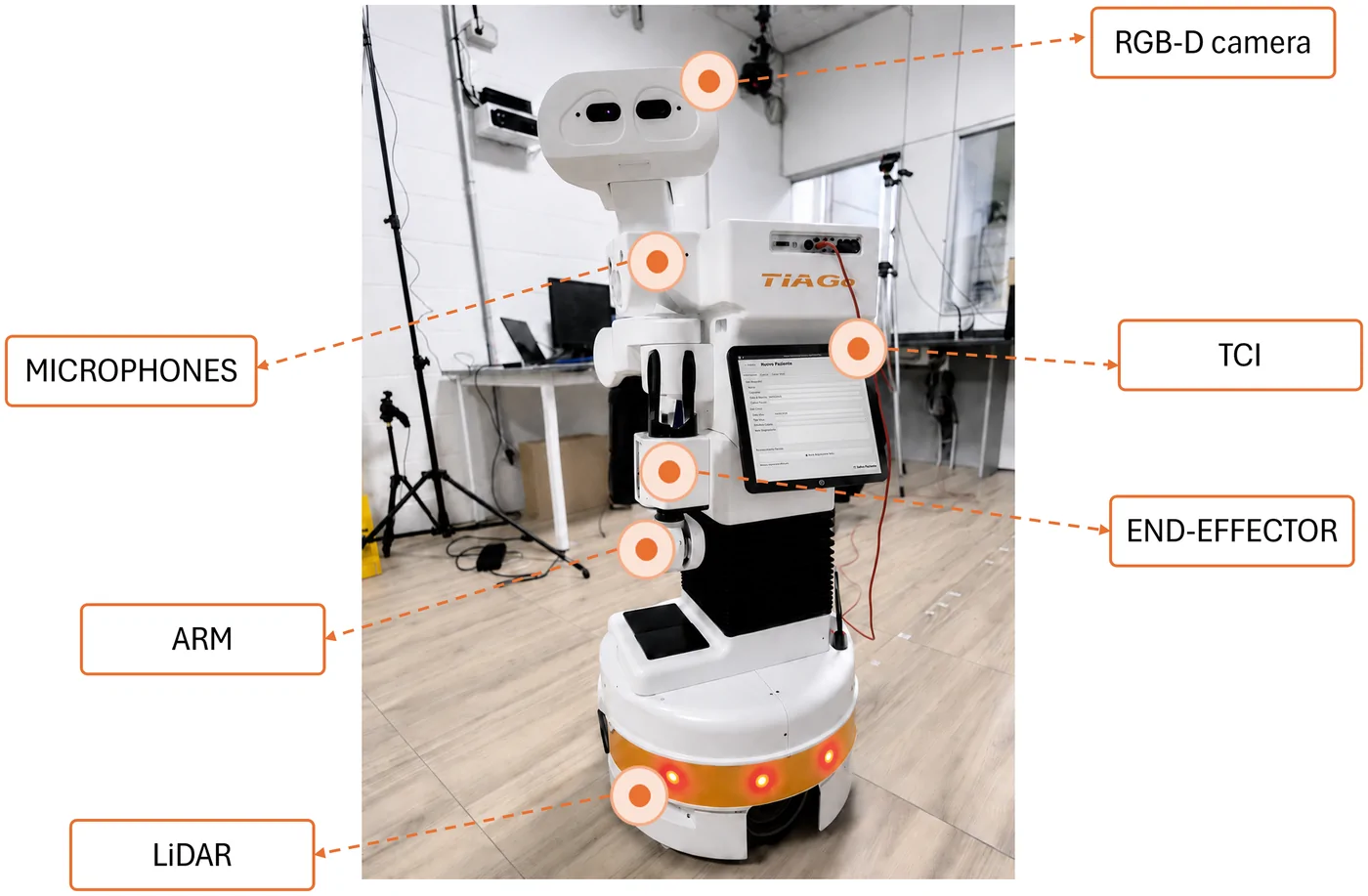
TIAGo robotic platform and main hardware components used in the proposed rehabilitation workflow.

At the software level, the system is organized into three main functional components: the TCI, the IM, and the EDM, integrated with a head-orientation monitoring subsystem (Figure 2). These components are described in the following sections, together with the main implementation details relevant to the proposed prototype.

**Figure 2:**
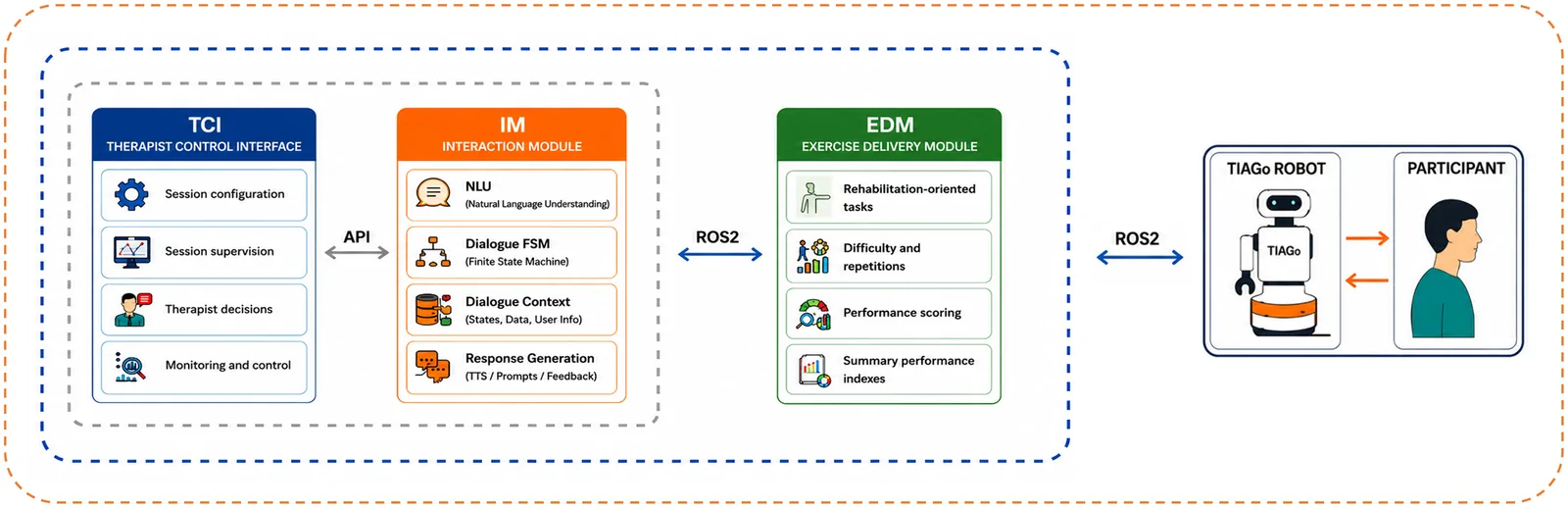
High-level architecture of the proposed ROS 2-based rehabilitation pipeline. The system is organized into three functional components: the Therapist Control Interface (TCI), the Interaction Module (IM), and the Exercise Delivery Module (EDM), integrated with attentional monitoring and robot-mediated participant interaction.

### 2.2 Therapist control interface

The TCI acts as the therapist-operated supervision and control layer of the pipeline (Figure 2). It receives structured events generated by the IM and by the robot-side modules, including session events, participant responses, task outcomes, attentional recall events, and safety-relevant notifications. Through the TCI, therapist supervision is embedded directly in the operational cycle of the system. The robot autonomously manages predefined low-risk interaction phases, whereas clinical or safety-relevant decisions are routed to the therapist. Safety-relevant or recovery-related events, including help requests, malaise reports, stop commands, repeated refusals, or repeated interaction failures, are escalated to the therapist. The therapist remains responsible for making high-level clinical decisions regarding continuation, repetition, interruption, or adaptation of the session.

The TCI was implemented as a modular Qt-based graphical interface using PyQt5 5.15.6. Representative screenshots of the TCI and screen-based task delivery are shown in Figure 3. The graphical interface was separated from the internal logic of the robot-side modules and from the IM. ROS 2 communication was handled by a dedicated interface layer, while asynchronous events were routed to the graphical interface through an internal event-dispatching mechanism. Therapist commands, exercise configuration updates, session-monitoring events, stop commands, dialogue outputs, and safety alerts were exchanged as structured events. This organization preserved interface responsiveness while keeping the GUI separated from the execution layer.

**Figure 3:**
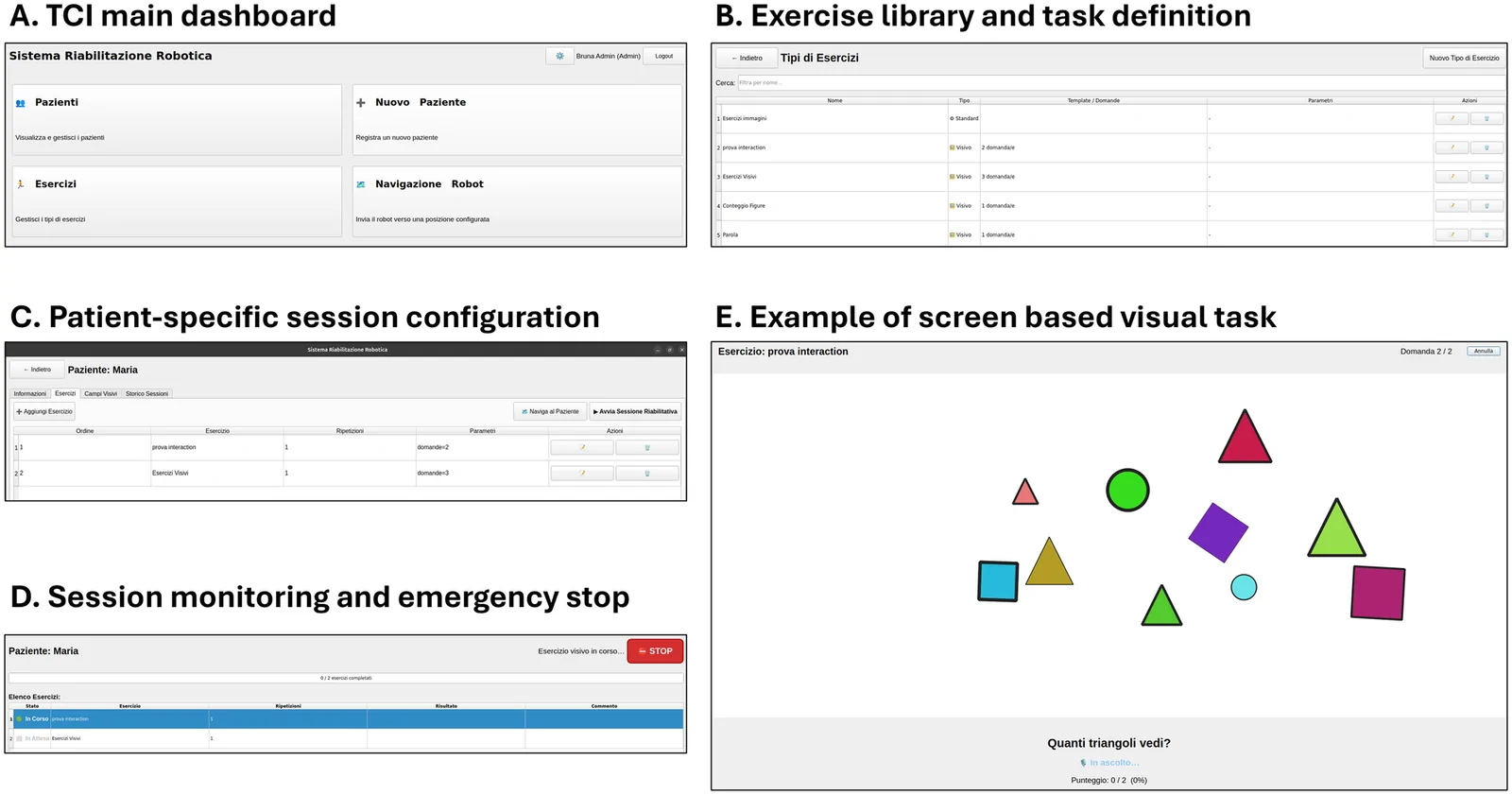
Therapist control interface and screen-based task delivery. Representative screenshots of the graphical interface used to configure, supervise, and administer the robotic rehabilitation workflow. **(A)** Main dashboard providing access to patient management, exercise management, and robot navigation. **(B)** Exercise library and task-definition interface used to manage the available rehabilitation-oriented exercises. **(C)** Patient-specific session configuration panel used to define the sequence of exercises and initiate robot navigation or session execution. **(D)** Session-monitoring interface showing the active exercise, progression status, and therapist-accessible emergency stop command. **(E)** Example of a screen-based visual task administered through the integrated TIAGo display.

### 2.3 Interaction module

The IM was designed as the central coordination layer between the participant, the robot, the TCI, and the EDM. It manages rehabilitation-oriented dialogue by interpreting participant inputs, controlling the interaction state, generating robot responses, and emitting structured events to the other components of the rehabilitation workflow. The IM was implemented as a Python-based dialogue core, decoupled from the graphical interface and from exercise-specific modules. The core dialogue logic was kept middleware-independent, while ROS 2/API adapters routed speech transcripts, graphical inputs, therapist commands, exercise events, dialogue outputs, safety-relevant notifications, and logging information among the TCI, the robot-side modules, and the EDM. This design allowed the FSM and NLU logic to be tested independently from the graphical interface and from individual exercise modules.

The IM follows a resource-aware and safety-aware logic. Speech transcripts, graphical inputs, therapist commands, and system events are first mapped by the NLU onto predefined workflow-relevant intents. These intents are then processed by the Dialogue FSM according to the current Dialogue Context, which stores the active state, pending confirmations, current exercise, previous prompts, and interaction counters. The FSM output is encoded as a structured Dialogue Output and transmitted through the ROS 2/API event-based communication layer to coordinate robot speech, graphical feedback, therapist-interface events, exercise-related notifications, and logging. The following subsections describe the main components of the IM: the NLU strategy, the Dialogue FSM, and the response-generation layer.

#### 2.3.1 Natural language understanding

The NLU module was implemented as a deterministic intent-recognition pipeline that converts heterogeneous inputs, including speech transcripts, graphical actions, therapist-interface commands, and system events, into canonical intents processed by the Dialogue FSM. The output of the NLU is a predefined intent label associated with the rehabilitation workflow. This rule-based design was selected to ensure low computational cost, transparent behavior, and predictable failure handling on an embodied robotic platform operating under resource and safety constraints. Speech transcripts were generated using an Italian ASR model within the Vosk offline speech-recognition toolkit (28), a toolkit developed within the Kaldi-based ASR ecosystem (29) and were then processed by the rule-based NLU pipeline. The ASR output was treated as an input transcript, and all clinically relevant decisions were made only after deterministic mapping onto the predefined intent set.

Speech-based inputs were first processed by a text-normalization layer. The normalizer converted text to lowercase, removed accents, punctuation marks, repeated spaces, and non-informative special characters, and standardized minor orthographic variations. This preprocessing reduced variability due to capitalization, punctuation, ASR formatting, and common transcription inconsistencies. For example, short affirmative utterances such as “Sì!”, “si”, or “SÌ.” were normalized to the same canonical form.

After normalization, the NLU applied a deterministic cascade of matching rules. Graphical actions, therapist-interface commands, and system events were mapped directly onto canonical intents or control events without linguistic interpretation. Speech inputs were then classified using exact matching for short and safety-relevant utterances, and keyword/rule-based or similarity-based matching for more variable expressions related to confirmation, refusal, pause, help, discomfort, confusion, task repetition, or stop requests.

The matching cascade was designed conservatively. Approximate matching was used only as a controlled recovery mechanism and only when the normalized input was sufficiently close to a predefined reference expression or contained clinically relevant lexical cues. The resulting similarity score was used as a rule-based acceptance criterion and was not interpreted as a calibrated probabilistic confidence measure. When the input was ambiguous, incomplete, or insufficiently reliable, the system avoided forcing an interpretation and assigned the utterance to an explicit fallback category, such as UNKNOWN or OUT_OF_CONTEXT.

This fallback behavior was deliberately explicit because incorrect interpretation may be more problematic than non-interpretation in a safety-relevant rehabilitation context. When a fallback category was assigned, the Dialogue FSM could ask for clarification, repeat the pending prompt, or increment the corresponding interaction counter. Repeated unknown, out-of-context, or unclear inputs were treated as possible indicators of interaction breakdown, fatigue, confusion, or communication difficulty and could trigger therapist escalation according to the FSM safety policy. The intent set included confirmations, refusals, pause requests, stop requests, help requests, malaise reports, confusion, repetition requests, lateral informational questions, unknown inputs, and out-of-context utterances. Semantically similar but clinically different utterances, such as fatigue, malaise, refusal, and stop requests, were kept as distinct categories because they require different safety responses and different events toward the therapist interface. This organization allowed the IM to preserve conversational flexibility while maintaining deterministic safety behavior.

The canonical intent set, representative utterances, matching priority, acceptance criteria, fallback categories, and escalation counters are reported in Supplementary Table S1.

#### 2.3.2 Dialogue FSM

The Dialogue FSM was designed to control the progression of the rehabilitation-oriented interaction through a finite set of explicit and clinically interpretable states. Its purpose is to ensure predictable session progression, explicit participant confirmation before relevant actions, preservation of the current interaction context, and controlled handling of safety-relevant events.

The main states implemented in the system include WAITING_SESSION_START, WAITING_START_CONFIRMATION, WAITING_EXERCISE_START, WAITING_EXERCISE_CONFIRMATION, EXERCISE_RUNNING, PAUSED, WAITING_THERAPIST_DECISION, CLOSING, and ERROR. Each state defines the expected inputs, admissible transitions, robot responses, and events emitted toward the other components of the rehabilitation pipeline. Session execution starts only after a command from the TCI and explicit participant confirmation. Similarly, each exercise is introduced by the robot and started only after participant confirmation. This double-confirmation mechanism was introduced to preserve participant autonomy and prevent the robot from initiating tasks without clear agreement.

The FSM distinguishes three main classes of input: progression events, state-preserving informational intents, and safety- or recovery-relevant events. Progression events include session start, participant confirmation, exercise introduction, exercise start, exercise result, therapist decision, and session closure. State-preserving informational intents include participant requests for contextual information, such as asking who the robot is, what exercise is being performed, why the task is required, how much time remains, or whether a pause is possible. These inputs are handled without changing the active state. For example, if the participant asks “*What should I do?*” while the system is waiting for exercise confirmation, the robot provides the task explanation and then returns to the pending confirmation prompt. Safety- or recovery-relevant events include malaise reports, help requests, stop commands, refusals, repeated unknown or out-of-context inputs, and out-of-sequence system events.

Counter-based recovery policies were implemented to distinguish isolated input failures from persistent interaction breakdowns. The counter limits were predefined during prototype development and applied consistently across sessions. A first refusal or temporary non-readiness generated reassurance and a reprompt, while a second consecutive refusal escalated the event to the therapist. In confirmation or dialogue-management states, three consecutive UNKNOWN or unresolved fallback inputs generated therapist escalation. These counters supported controlled recovery from occasional recognition or interpretation failures while preventing autonomous continuation during persistent interaction breakdowns. Temporary interruptions moved the system to PAUSED, where the current exercise and dialogue context were preserved. Short voluntary pauses could be resolved through participant confirmation, while malaise reports, repeated confusion, repeated refusals, stop requests, or other safety-relevant events redirected the FSM to WAITING_THERAPIST_DECISION. In this protected state, the robot did not autonomously resume the rehabilitation workflow but emitted a structured alert to the TCI and waited for an explicit therapist decision, such as resume, repeat, skip, or terminate.

The Dialogue Context supports FSM operation by storing the current state, current exercise, last prompt, pending confirmation, previous state, participant responses, and counters related to repeated refusals, unknown inputs, out-of-context utterances, silence events, help requests, and malaise reports. These variables were maintained during the interaction and recorded in a structured JSON-based log database. This allowed the system to distinguish isolated interaction failures from repeated patterns that may indicate fatigue, confusion, disengagement, or communication breakdown.

The complete state-transition matrix, including admissible inputs, emitted outputs, next states, ignored-event conditions, and counter-based escalation criteria, is provided in Supplementary Table S2. Figure 4 summarizes the main FSM structure and illustrates the recovery paths used for progression events, state-preserving questions, task-specific utterances, safety-relevant events, and therapist-mediated decisions.

**Figure 4:**
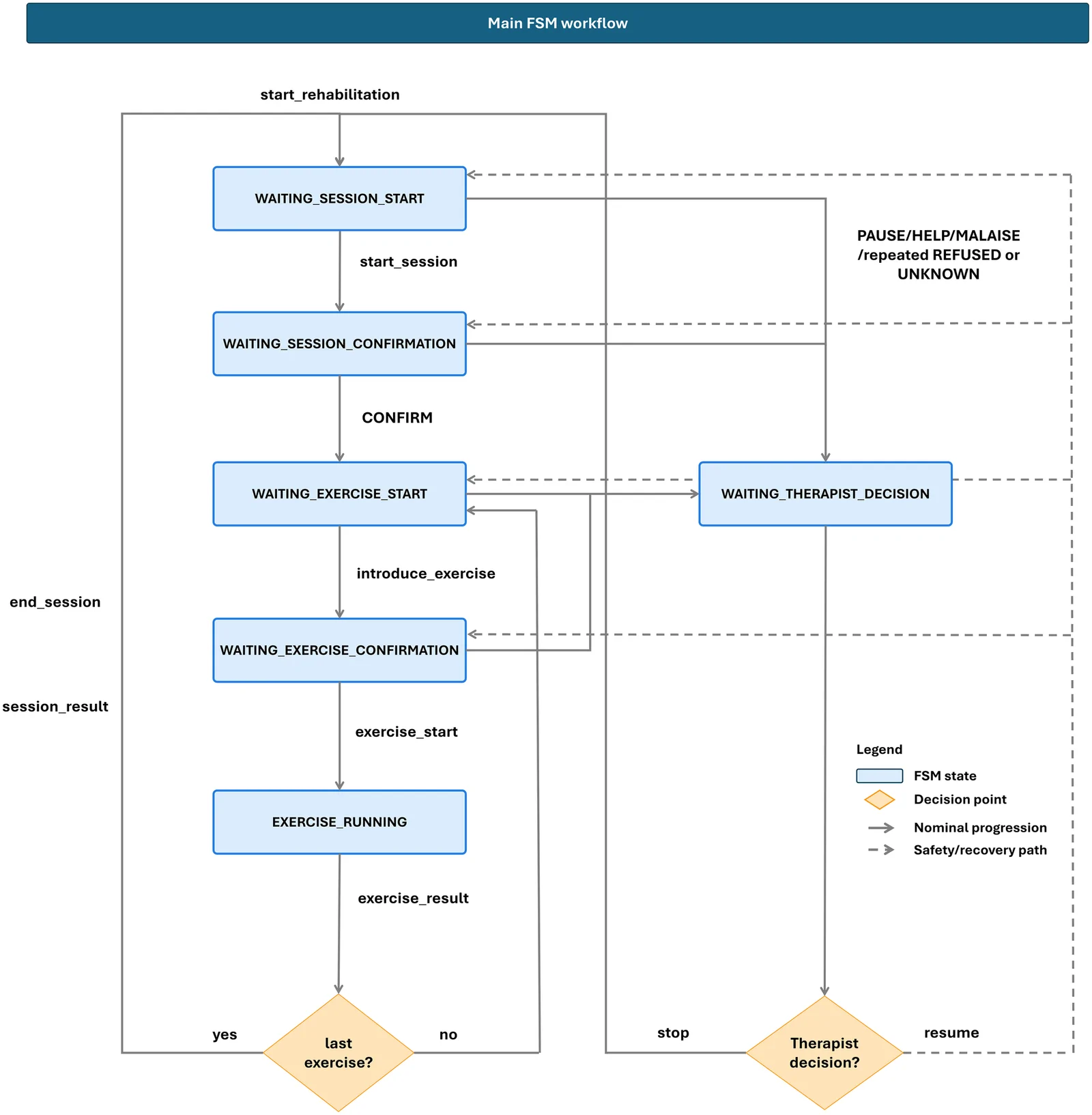
Main workflow of the therapist-supervised dialogue FSM. Light-blue rectangles represent FSM states, yellow diamonds represent decision points, solid arrows indicate nominal workflow progression, and dashed arrows indicate safety/recovery or therapist-mediated return paths. The nominal workflow proceeds from session initialization to exercise confirmation, exercise execution, and session closure. The “last exercise?” decision determines whether the system proceeds to the next exercise or closes the session. Safety- or recovery-relevant events redirect the FSM to WAITING_THERAPIST_DECISION, where the system waits for an explicit therapist decision before resuming, repeating, skipping, or terminating the session. If continuation is approved, the FSM returns to the previously active state; otherwise, the session is closed and the workflow returns to WAITING_SESSION_START. State-preserving informational requests, such as “*Who are you?*”, “*What are we doing today?*”, or “*What should I do?*”, are answered without changing the current FSM state and are therefore not represented as separate transitions.

At each transition, the FSM produces a structured result exposed through the API/event-based communication layer. This result may include the updated state, the robot utterance, graphical-interface information, events for the TCI, notifications for the EDM, and logging metadata. In this way, dialogue state, robot behavior, therapist supervision, and exercise delivery remain synchronized without direct coupling between their internal implementations.

#### 2.3.3 Response generation and dialogue output

The response-generation layer converts the decision produced by the Dialogue FSM into structured outputs that can be executed by the different components of the rehabilitation pipeline. Its role is to separate dialogue control from output execution: the FSM determines what should happen next, whereas the response-generation layer defines how this decision is expressed through speech, graphical feedback, therapist-interface events, exercise-related notifications, and logging metadata.

Robot utterances are selected from a configurable set of predefined responses, rather than being hard-coded inside the FSM. This allows confirmation requests, exercise explanations, clarification prompts, motivational messages, pause confirmations, and safety-related utterances to be modified without changing the dialogue-control logic. Exercise-specific prompts are handled separately from general session-level responses, allowing task-related instructions and feedback to be adapted independently from the FSM. Robot verbal output was generated using Acapela text-to-speech voices (30). The TTS layer executed the utterances selected by the response-generation layer, without modifying the dialogue policy, the selected response key, or the FSM transition logic.

Each interaction step produces a structured Dialogue Output object, which may include the robot utterance, graphical-interface updates, updated dialogue state, TCI events, EDM notifications or commands, safety alerts, and logging metadata. The Dialogue Output is exposed through the API and event-based communication layer rather than being directly coupled to a specific actuator, GUI component, or ROS 2 node. This organization allows speech synthesis, graphical updates, exercise commands, therapist notifications, and logging procedures to be modified or tested independently from the dialogue-control logic.

### 2.4 Exercise delivery module

The EDM was implemented within the ROS 2 architecture as the component responsible for coordinating the rehabilitation-oriented tasks included in the robotic workflow. It receives exercise configuration parameters from the TCI, activates the selected task, monitors task execution, collects task-related outcomes, and returns structured performance information to the rest of the pipeline.

Exercise-related messages included, when applicable, the exercise identifier, exercise type, task name, number of repetitions, difficulty level, task-specific parameters, completion status, percentage score, execution time, and structured result payload. These messages allowed the TCI, EDM, and IM to synchronize exercise start, exercise completion, score reporting, and therapist-facing recommendations without embedding exercise-specific logic inside the dialogue controller.

In the proposed architecture, the EDM is not a monolithic exercise implementation, but a coordination layer interfacing with different exercise-specific modules. Depending on the task, execution may involve the robotic arm and end-effector, the integrated screen, speech-based interaction, auditory feedback, or attentional monitoring. This organization allows the same pipeline to support robot-kinematics mediated exercises, screen-based visual tasks, and multimodal exercises combining robot motion, visual observation, speech input, and feedback.

For each exercise, the EDM can compute or receive task-specific summary indices, including response accuracy, percentage score, number of completed repetitions, execution time, response timing, verbal attempts, attentional recall events, and ASR-related mismatches. These outputs provide quantitative information on task execution and interaction quality, supporting structured logging, therapist supervision, and subsequent analysis.

A rule-based progression logic was included to support transparent interpretation of task outcomes. Scores equal to or above 75% were interpreted as successful task completion and could support progression or increased difficulty, whereas scores below 25% suggested that the exercise should be repeated in a simplified form. Intermediate scores between 25% and 74% indicated partial completion and could support repetition at the same difficulty level or therapist-mediated adjustment. This approach is consistent with rehabilitation-oriented serious games and exergames, where task difficulty is commonly adapted according to user performance to maintain an appropriate level of challenge and support therapist-supervised personalization (31–33).

The EDM is synchronized with the attentional monitoring subsystem so that head-orientation metrics and attention-recall events can be collected together with task outcomes. This allows the system to support interpretation of task performance in relation to head-orientation toward the robot-facing interaction area, fatigue, or reduced engagement during the exercise. In this way, the EDM contributes both to task delivery and to quantitative assessment within the rehabilitation workflow.

### 2.5 Attentional monitoring subsystem

The attentional monitoring subsystem was designed to provide a continuous estimate of the participant's head orientation relative to the robotic platform during interaction and exercise phases. This estimate was implemented using the RGB-D sensor mounted on TIAGo. Face tracking and facial landmark extraction were performed in real time using MediaPipe-based processing, implemented with MediaPipe 0.10.9 and OpenCV 4.10.0.84. These libraries were used to support real-time facial landmark detection and head-orientation computation from RGB-D input (34, 35), within the modular TIAGo-based rehabilitation platform previously developed for hemispatial inattention (23).

From the facial landmarks detected, the system computed the participant's head orientation relative to the robot. The horizontal and vertical components of head rotation were combined into a single angular value representing the overall deviation from the robot-facing direction. Lower values indicated orientation toward the robot, whereas higher values indicated increasing deviation. This measure was used as an operational head-orientation metric during robotic interaction. During the session, the combined head-orientation angle was compared with a predefined operational threshold. In the implemented configuration, the threshold was set to 50° and was used to identify large deviations of the head away from the robot-facing interaction area. This relatively wide threshold was selected to tolerate natural head movements during verbal interaction and to account for the estimation error observed in previous stereophotogrammetric validation of the head-orientation module within the TIAGo-based rehabilitation platform (22). The threshold was therefore used as a pragmatic trigger for adaptive auditory recall within the prototype workflow.

When the combined head-orientation angle exceeded this threshold, an attention-related event was generated and transmitted to the IM and EDM, allowing TIAGo to produce an adaptive auditory prompt aimed at recalling the participant's orientation toward the robot. All attention-related events were logged together with head-orientation metrics, recall timing, and recall outcomes when available. In the target clinical scenario, this subsystem is intended to support attention-oriented rehabilitation in patients with hemispatial inattention by encouraging orientation toward the relevant hemispace. In the present healthy-participant evaluation, it was used to test the feasibility and stability of real-time head-orientation monitoring and adaptive auditory recall within the multimodal workflow.

### 2.6 Experimental protocol and task delivery

Each experimental session followed a structured sequence designed to reproduce the planned therapist-supervised rehabilitation workflow under controlled conditions. The protocol integrated therapist-mediated configuration, autonomous robot positioning, speech-based interaction, attentional monitoring, robot-mediated task execution, screen-based visual exercises, structured logging, and final usability assessment.

At the beginning of each session, the therapist configured the session through the TCI by selecting the participant and defining the exercises to be administered. In the present experimental protocol, these exercises followed the predefined sequence described above. After session initialization, TIAGo autonomously navigated toward the participant and reached the predefined interaction position. In the target clinical scenario, this positioning strategy is intended to place the robot on the side selected for attentional stimulation, typically corresponding to the neglected hemispace. The robot's physical presence, voice, screen, arm, and end-effector are therefore located in a defined spatial region, encouraging orientation toward the robotic platform before explicit task execution begins. Once positioned, the robot initiated verbal engagement through speech synthesis and speech recognition. During the interaction, the attentional monitoring subsystem continuously estimated the participant's head orientation.

After the initial engagement phase, the participant performed the end-effector recognition task, designed as a motor-observational and speech-mediated exercise. TIAGo opened or closed its gripper and verbally asked the participant to identify the current configuration of the robotic hand. The participant's response was acquired through speech recognition, and the robot provided immediate auditory feedback on response correctness. For each acquisition, the task consisted of three interaction cycles per participant, resulting in 48 end-effector recognition cycles per acquisition across the 16 participants. The EDM recorded response correctness, number of verbal attempts, timing information, task outcome, and head-orientation-related metrics.

Subsequently, each participant completed a predefined dialogue/questionnaire interaction composed of 15 verbal questions. Although this phase was not an exercise in the strict motor sense, it was included in the protocol to assess speech-based communication, maintenance of orientation toward the robot during repeated verbal interaction, ASR-related interaction events, and structured logging under repeated conversational conditions. During this phase, the system acquired participant responses, monitored head orientation, generated attention-recall prompts when required, and logged the timing and outcome of each interaction event. With 16 participants, this resulted in 240 dialogue/questionnaire interaction events per acquisition.

The protocol also included monitor-based visual tasks displayed on the integrated TIAGo screen. These tasks consisted of an image-recognition exercise and a geometric shape-counting exercise, both aimed at stimulating visual exploration, sustained orientation toward the robot-facing interaction area, and interaction with the robotic platform. Each screen-based task included five items and was delivered once per participant during each acquisition. During these activities, the EDM recorded execution time, verbal responses, interaction timing, percentage scores, task-completion statistics, item-level information, and head-orientation-related metrics.

At the end of each acquisition, participants completed usability and acceptance questionnaires. The same protocol was repeated after 1 month to explore whether the integrated workflow could reproduce comparable interaction dynamics, task-related outcomes, attentional monitoring metrics, ASR-related events, and user-perceived usability across repeated exposure.

### 2.7 Participants and experimental setting

Sixteen healthy participants were enrolled in a preliminary feasibility and usability evaluation with two acquisitions separated by 1 month. The sample included 7 males and 9 females, with a mean age of 54.4 ± 6.8 years. Healthy participants were selected to assess system stability, interaction flow, safety-aware interaction management, data acquisition, and acceptability under control conditions before future experimentation with neurological patients. All procedures involving human participants were conducted in accordance with the principles of the Declaration of Helsinki. All participants provided written informed consent before taking part in the experimental acquisitions. Data was anonymized before analysis, and no sensitive personal or clinical information was used for the purposes of this study. The analyses were based on anonymized interaction logs, questionnaire scores, task outcomes, speech-recognition outputs, attention-recall events, and head-orientation metrics.

All experimental sessions were conducted in the Living Lab of the Centre of Excellence on Rehabilitation Devices and Digital Instruments (CoE REDI). The environment was arranged to reproduce indoor rehabilitation conditions, including bedside and wheelchair-oriented interaction scenarios. The same controlled laboratory setting and comparable acoustic and spatial conditions were maintained across acquisitions. During the interaction, TIAGo was positioned laterally with respect to the participant, according to the planned rehabilitation configuration in which the spatial position of the robot is intended to act as an attentional cue toward the neglected hemispace in future clinical use. Speech was acquired through the onboard microphones of the TIAGo platform, without additional external microphones.

### 2.8 Data collection and outcome measures

During each session, the robotic framework automatically recorded multimodal interaction data, including speech events, participant verbal responses, graphical and task-related events, response timing, exercise outcomes, attention-recall events, and estimated head-orientation angles. These data were stored as timestamped session logs in a structured JSON-based database generated by the local logging module of the robotic framework. Each logged event included, when applicable, the session identifier, participant identifier, current FSM state, recognized intent, confidence or similarity score, exercise identifier and type, task score, therapist alert type, timestamp, and session metadata. Out-of-sequence events, such as exercise results received before an exercise was running or duplicate session-closure events, were also stored as ignored events.

Task-related outcomes were extracted according to the administered activity. End-effector recognition was quantified in terms of response correctness, number of cycles, verbal attempts, task outcome, and potential ASR-related mismatches. Monitor-based visual tasks were quantified in terms of completion status, percentage score, execution duration, item-level information, and task-completion statistics. Dialogue/questionnaire interactions were analyzed in terms of participant responses, interaction timing, attention-recall events, and speech-recognition outputs.

The attentional monitoring subsystem was evaluated using operational head-orientation metrics, including attention-recall attempts, head-monitoring attempts, mean combined head-orientation angle, threshold-crossing events, and consistency of head orientation before and after verbal responses. These metrics were used to quantify orientation toward the robot-facing interaction area rather than direct visual attention or neglect-specific exploratory behavior.

ASR outputs were analyzed within the same robot-centered acoustic and spatial configuration used during the complete multimodal interaction workflow. The analysis focused on interaction events relevant to task execution, response interpretation, dialogue progression, and FSM-based interaction management. This approach was consistent with the structure of the experimental protocol, which included both constrained task responses with expected answers and dialogue/questionnaire responses without fixed lexical targets.

Two ASR-related categories were defined. In the end-effector recognition task, each participant response was associated with an expected answer, corresponding to the Italian responses “aperta” and “chiusa” for the open and closed gripper configurations. A response was classified as correct when the ASR transcript matched the expected answer or an accepted equivalent form. When an incorrect transcript contained a fragmented, incomplete, or phonetically similar form of the expected answer, the event was classified as a probable ASR-related mismatch. Examples included fragmented recognition of “chiusa” as “chi usa” or “usa”, and phonetic substitution of “aperta” with acoustically similar words. Other incorrect responses were retained as task-level incorrect responses.

For the dialogue/questionnaire phase, responses were analyzed without a fixed lexical target. ASR outputs were classified as potentially suspicious when they were fragmented, incomplete, phonetically implausible, semantically inconsistent with the question context, or too short to support reliable interpretation. These events were counted as ASR-related interaction vulnerabilities and used to assess whether speech-recognition issues affected the continuity of the multimodal workflow.

All ASR classifications were based on structured interaction logs, task context, expected answers when available, and ASR transcripts generated during the experimental sessions. The resulting metrics were reported as interaction-level ASR-related event rates and were used to describe the impact of ASR behavior on task execution, dialogue progression, and workflow continuity.

### 2.9 Usability and acceptance assessment

At the end of each experimental session, participants completed a usability and acceptance assessment battery including the SUS, the UMUX-Lite, and selected constructs from the UTAUT. The SUS was used to assess the perceived usability of the robotic platform, while the UMUX-Lite provided a concise complementary measure of perceived usefulness and ease of use (24, 25).

The UTAUT-based assessment was used to investigate acceptance-related dimensions, including perceived usefulness, ease of use, trust, sociability, adaptability, anxiety toward robotic systems, perceived utility in rehabilitation scenarios, and intention to use the platform. The selection of UTAUT constructs was based on their relevance for evaluating user acceptance of interactive technologies and robotic systems in healthcare-oriented contexts (26, 27). Because selected UTAUT-related dimensions were used, the exact Italian item wording, construct grouping, response scale, and scoring direction are reported in Supplementary Table S3. The questionnaire also included three preliminary items on participants' familiarity with computers and Internet use. UTAUT-related items were rated on a five-point Likert scale ranging from “totally disagree” to “totally agree”; anxiety-related items were interpreted separately because higher scores indicate greater fear, discomfort, or apprehension toward the robotic platform.

### 2.10 Data analysis

The acquired multimodal interaction data were analyzed to evaluate technical robustness, workflow reproducibility, and usability of the robotic framework. Descriptive statistics were computed for the main outcome measures, including end-effector recognition accuracy, verbal interaction attempts, head-monitoring attempts, mean head-orientation angle, exercise duration, task-completion scores, attention-recall events, and questionnaire scores.

Given the preliminary nature of the study, the limited sample size, and the repeated-measures structure of the experimental protocol, non-parametric statistical methods were adopted. Paired comparisons between repeated acquisitions were performed using Wilcoxon signed-rank tests to evaluate differences in end-effector recognition accuracy, verbal interaction stability, head-monitoring attempts, head-orientation estimates, and usability-related scores. When more than two repeated interaction cycles or task repetitions were compared within the same session, Friedman tests were used to investigate intra-session trends across consecutive cycles.

Spearman rank correlation coefficients were computed between head-orientation measurements acquired before and after participant verbal responses to evaluate the consistency of orientation toward the robot-facing interaction area during interaction. One-month workflow reproducibility was assessed by comparing workflow completion, task-related outcomes, head-orientation monitoring metrics, ASR-related interaction events, and usability scores between the first acquisition and the retest. Test–retest stability was additionally explored for selected technical and questionnaire-related metrics using intraclass correlation as supportive indicators of component-level stability.

Screen-based visual task outcomes were analyzed separately for the image-recognition and geometric shape-counting exercises. For each task and acquisition, completion status, percentage score, execution duration, number of administered items, item-level prompts, and expected answers were extracted from the exercise logs. Only valid participant sessions were retained for this analysis. Descriptive statistics were computed for task-completion rate, percentage score, and execution duration at test and retest.

ASR outputs were analyzed using the interaction-event classification protocol described above. For the constrained end-effector recognition task, ASR-related events were classified by comparing the transcript with the expected response and by identifying fragmented, incomplete, or phonetically similar forms suggestive of recognition mismatch. For the dialogue/questionnaire phase, where no fixed lexical ground truth was available, suspicious outputs were identified when transcripts were fragmented, incomplete, phonetically implausible, semantically inconsistent with the question context, or too short to support reliable interpretation, and were interpreted as ASR-related interaction vulnerabilities rather than as formal word-level ASR errors.

## 3 Results

### 3.1 Operational feasibility of the integrated workflow

The operational feasibility of the integrated robotic workflow was assessed across the first acquisition and the 1-month retest. All 16 healthy participants completed both experimental acquisitions under controlled Living Lab conditions. In both sessions, the system supported the complete sequence of therapist-mediated session configuration, autonomous robot navigation, predefined positioning, speech-based engagement, head-orientation monitoring, end-effector recognition, dialogue/questionnaire interaction, monitor-based visual tasks, structured data logging, and usability assessment.

Both acquisitions generated complete datasets, including 16 experimental sessions, 48 end-effector recognition cycles, and 240 dialogue/questionnaire interaction events per acquisition. Across test and retest, the ROS 2-based architecture maintained synchronization among the TCI, the IM, the EDM, the attentional monitoring subsystem, and the robot execution layer.

No adverse events or safety-relevant events requiring interruption of the experimental workflow were observed during either acquisition. TIAGo completed the planned interaction sequence, executed the selected tasks, and recorded multimodal interaction data throughout the sessions. Attention-related events, when detected, were managed through adaptive auditory prompts and logged without disrupting the overall workflow.

### 3.2 Attentional monitoring and adaptive recall

The attentional monitoring subsystem operated continuously during both acquisitions and provided real-time head-orientation estimates during the interaction. In the first acquisition, participants generally required only one head-orientation monitoring attempt, with an average number of attempts of 1.08 ± 0.24 during the first repetition and 1.00 ± 0.00 during the second repetition. In the retest acquisition, head-orientation monitoring attempts remained stable at 1.00 ± 0.00 in both repetitions.

Mean head-orientation angles during the first acquisition were 18.74 ± 9.77° in the first repetition and 11.15 ± 4.85° in the second repetition. In the retest acquisition, the corresponding values were 17.03 ± 4.70° and 18.27 ± 7.01° in the first and second repetitions, respectively. These values indicate that participants were generally detected as oriented toward the robot-facing interaction area according to the operational head-orientation criterion.

During the dialogue/questionnaire phase, attention-recall events were infrequent. In the first acquisition, 5 recall events were generated across 240 analyzed dialogue/questionnaire interactions, corresponding to 2.1% of the total events. In the retest acquisition, 7 recall events were generated across 240 interactions, corresponding to 2.9%. Recall events were sparsely distributed across the dialogue sequence, and no significant effect of question position was observed in either acquisition. The Friedman test showed no significant question-order effect in the first acquisition, *χ*^2^ = 10.606, *p* = 0.7167, or in the retest acquisition, *χ*^2^ = 12.809, *p* = 0.5417.

Head-orientation measurements before and after verbal responses were analyzed to assess head-orientation stability during dialogue. In the first acquisition, pre- and post-response values showed a strong correlation (Spearman *r* = 0.909, *p* < 0.001, *n* = 240), with limited mean variation (Δ = 0.34 ± 3.33°) and no significant pre/post difference (Wilcoxon *W* = 12,371.000, *p* = 0.2214). In the retest, the correlation was weaker but significant (Spearman *r* = 0.229, *p* = 0.0004, *n* = 239), with limited mean variation (Δ = 0.69 ± 10.35°) and no significant pre/post difference (Wilcoxon *W* = 12,856.500, *p* = 0.3901). Participant-level attention-recall rates and head-orientation variations are summarized in Figures 5A,B.

**Figure 5:**
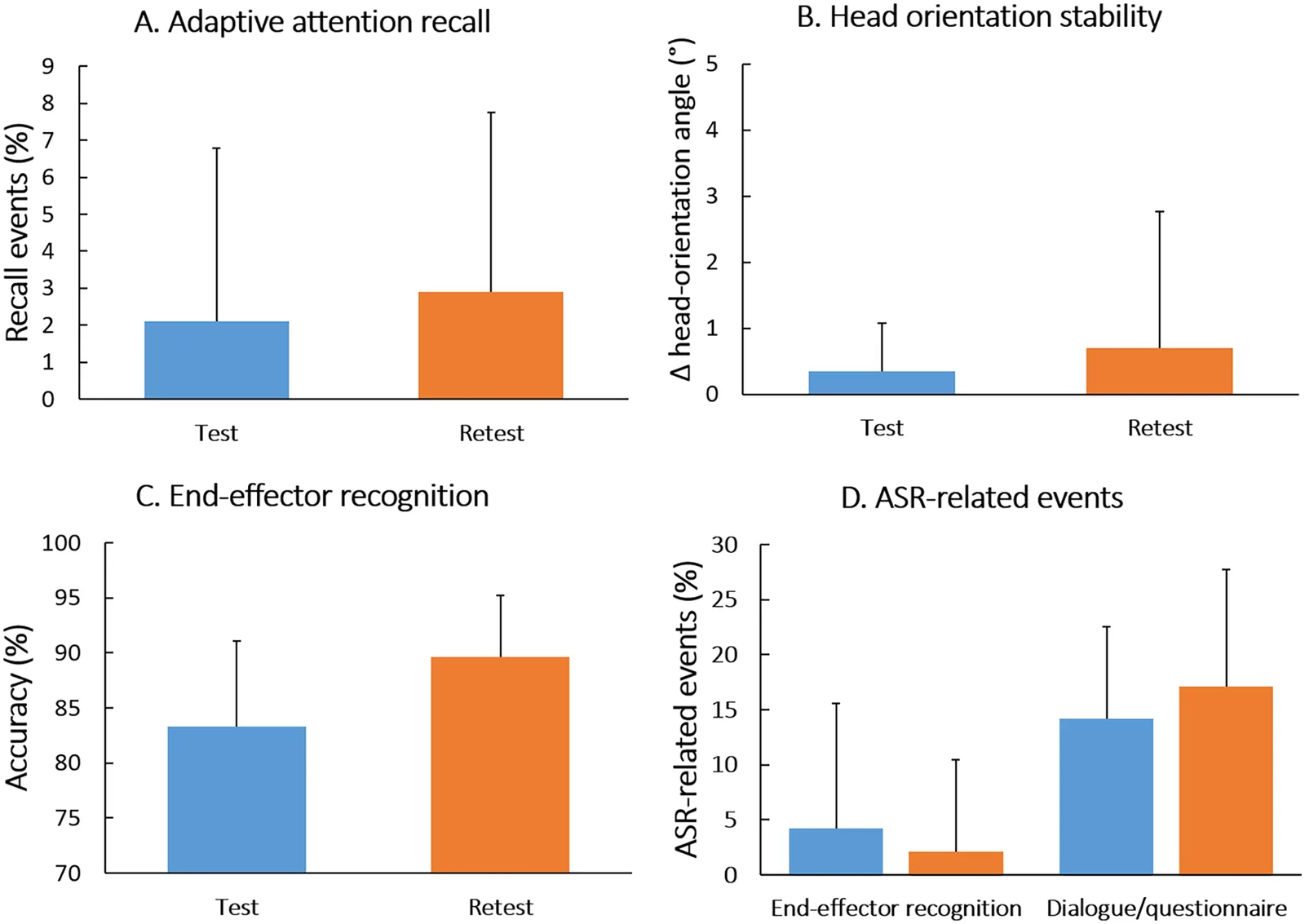
Technical and interaction robustness across test and retest acquisitions. Bars report participant-level mean ± standard deviation. **(A)** Percentage of attention-recall events during dialogue/questionnaire interactions. **(B)** Participant-level variation in head-orientation angle before verbal responses. **(C)** End-effector recognition accuracy. **(D)** ASR-related event rates for the end-effector recognition task and the dialogue/questionnaire interaction.

### 3.3 End-effector recognition task and verbal interaction

The end-effector recognition task was used to evaluate the synchronization of robot action, visual observation, speech-based response acquisition, and auditory feedback. During the first acquisition, participants completed 48 interaction cycles, with 40 correct responses, corresponding to an overall recognition accuracy of 83.3%. In the retest acquisition, participants completed 48 interaction cycles, with 43 correct responses, corresponding to an overall accuracy of 89.6%. In the participant-level graphical summary, end-effector recognition accuracy was 83.3 ± 7.8% at test and 89.6 ± 5.6% at retest (Figure 5C).

Cycle-level analysis showed no significant variation in task performance across consecutive repetitions. In the first acquisition, mean correctness values were 81% for the first cycle, 94% for the second cycle, and 75% for the third cycle. No significant difference was observed across cycles, Friedman test: *χ*^2^ = 1.750, *p* = 0.4169. In the retest acquisition, mean correctness values were 81%, 100%, and 88% for the first, second, and third cycles, respectively, again with no significant cycle effect, Friedman test: *χ*^2^ = 2.800, *p* = 0.2466.

When repeated interaction blocks were compared within each acquisition, end-effector recognition accuracy did not differ significantly. In the first acquisition, mean accuracy was 0.92 ± 0.15 in the first repetition and 0.75 ± 0.15 in the second repetition, Wilcoxon signed-rank test: *W* = 3.5, *p* = 0.2188. In the retest acquisition, mean accuracy was 0.92 ± 0.15 and 0.88 ± 0.17 in the first and second repetitions, respectively, with no significant difference between repetitions, Wilcoxon signed-rank test: *W* = 6.0, *p* = 1.0.

The verbal interaction pipeline also showed consistent operational behavior during the end-effector recognition task. In the first acquisition, participants required approximately one verbal attempt per cycle, with mean values of 1.04 ± 0.12 attempts in the first repetition and 1.00 ± 0.00 in the second repetition. In the retest acquisition, the corresponding values were 1.00 ± 0.00 and 1.08 ± 0.15. No significant difference between repetitions was observed in either acquisition. These results indicate that the system was generally able to acquire participant responses without repeated prompting, supporting the operational reliability of the speech-mediated task execution pipeline under the tested conditions.

### 3.4 Screen-based visual tasks

Screen-based visual tasks were included to evaluate the ability of the pipeline to administer TCI-mediated exercises through the integrated TIAGo screen while recording task completion, performance scores, execution timing, and item-level information. Two visual tasks were administered: an image-recognition task and a geometric shape-counting task. Each task included five items and was delivered once per participant during each acquisition.

All 16 participants completed both screen-based tasks at test and retest, corresponding to 32 completed screen-based task executions per acquisition. In the image-recognition task, the mean score was 63.75 ± 25.53% at test and 67.50 ± 26.20% at retest. The median score was 60.0% at test and 80.0% at retest, with scores ranging from 20% to 100% in both acquisitions. Mean execution duration was 32.13 ± 2.13 s at test and 43.56 ± 1.97 s at retest.

In the shape-counting task, the mean score was 61.25 ± 23.63% at test and 88.75 ± 16.28% at retest. The median score increased from 60.0% at test to 100.0% at retest, with scores ranging from 20% to 100% at test and from 60% to 100% at retest. Mean execution duration was 43.38 ± 4.81 s at test and 53.44 ± 4.77 s at retest.

### 3.5 ASR-related interaction events

ASR outputs were inspected to identify recognition mismatches, fragmented utterances, phonetic ambiguities, incomplete decoding, and potentially suspicious transcriptions. In the end-effector recognition task, in which the expected verbal responses were constrained to the Italian words corresponding to “open” and “closed”, incorrect task responses were further inspected to identify probable ASR-related errors. During the first acquisition, 8 out of 48 end-effector recognition responses were classified as incorrect, corresponding to 16.7% of responses. Among these, 2 responses, corresponding to 4.2% of all end-effector task responses, were identified as probable ASR-related errors. In the retest acquisition, 5 out of 48 responses were classified as incorrect, corresponding to 10.4%, and 1 response, corresponding to 2.1%, was identified as a probable ASR-related error.

The ASR-related events observed in the end-effector recognition task were mainly associated with short verbal responses, partial recognitions, and phonetic ambiguities in the expected response words.

In the dialogue/questionnaire phase, responses were analyzed without fixed expected lexical answers. ASR outputs were classified as potentially suspicious when they were fragmented, incomplete, phonetically implausible, too short to support reliable interpretation, or semantically inconsistent with the question context. In the first acquisition, 34 potentially suspicious ASR outputs were identified across 240 responses, corresponding to 14.2% of analyzed interactions. In the retest acquisition, 41 suspicious outputs were identified across 240 responses, corresponding to 17.1%. Most suspicious outputs consisted of short or fragmented utterances.

The interaction workflow remained operational despite these ASR-related events, and the system continued to support multimodal dialogue, head-orientation monitoring, structured logging, and task progression throughout the sessions. Figure 5D summarizes participant-level ASR-related event rates, with probable ASR-related errors in the end-effector recognition task equal to 4.2 ± 11.4% at test and 2.1 ± 8.3% at retest, and suspicious ASR outputs during the dialogue/questionnaire phase equal to 14.2 ± 8.4% at test and 17.1 ± 10.6% at retest.

### 3.6 Usability and acceptance

Usability and acceptance were assessed using SUS, UMUX-Lite, and selected UTAUT-related constructs. Participant-reported usability and acceptance outcomes are summarized in Figure 6.

**Figure 6:**
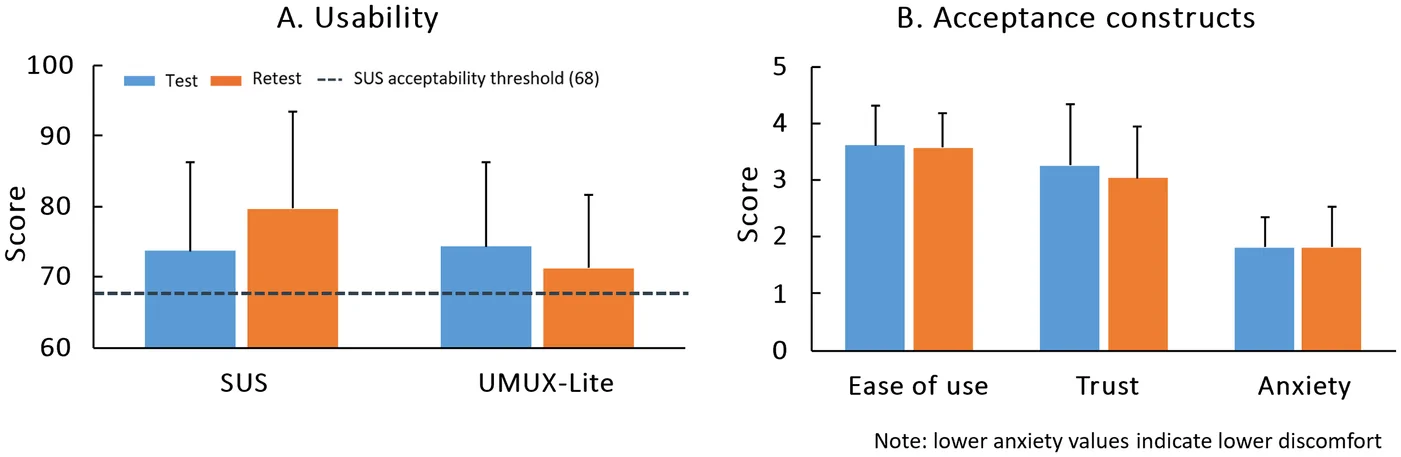
Usability and acceptance outcomes across test and retest acquisitions. **(A)** SUS and UMUX-Lite scores. The dashed horizontal line indicates the commonly adopted SUS acceptability threshold of 68. **(B)** Selected UTAUT constructs, including ease of use, trust, and anxiety. Data are reported as mean ± standard deviation across participants. Lower anxiety scores indicate lower discomfort or apprehension during interaction with the robotic platform.

In the first acquisition, the mean SUS score was 73.75 ± 12.59, with a median of 77.5 and a range from 45 to 90. In the retest acquisition, the SUS score increased to 79.69 ± 13.81, with values ranging from 50 to 97.5. Both acquisitions therefore showed mean SUS values above the commonly adopted acceptability threshold of 68, indicating positively perceived usability of the robotic rehabilitation workflow.

UMUX-Lite scores also supported a favorable usability profile. In the first acquisition, the standardized UMUX-Lite score was 74.36 ± 11.97, with a median of 77.07 and a range from 49.98 to 87.90. In the retest acquisition, the standardized UMUX-Lite score was 71.31 ± 10.37, with values ranging from 49.98 to 87.90. These results indicate that participants generally perceived the system as usable and understandable across repeated exposure.

The UTAUT-based analysis provided additional information on acceptance-related dimensions. In the first acquisition, Ease of Use reached 3.61 ± 0.69, Trust reached 3.25 ± 1.09, and Anxiety remained low at 1.82 ± 0.54. In the retest acquisition, the corresponding scores were 3.57 ± 0.60 for Ease of Use, 3.04 ± 0.91 for Trust, and 1.82 ± 0.72 for Anxiety. These values indicate that participants perceived the platform as relatively easy to use, with moderate-to-positive trust and limited anxiety during interaction with the robot.

SUS and UMUX-Lite scores are reported in Figure 6A, while selected UTAUT constructs are shown in Figure 6B.

### 3.7 One-month workflow reproducibility of the integrated workflow

The 1-month retest was used to evaluate the reproducibility of workflow execution. Across the two acquisitions, participants completed the same sequence of therapist-mediated session configuration, autonomous robot navigation, speech-based engagement, head-orientation monitoring, end-effector recognition, dialogue/questionnaire interaction, screen-based visual tasks, structured data logging, and usability assessment. The complete interaction sequence was successfully re-executed across the 1-month interval, supporting the reproducibility of the integrated robotic workflow under supervised preclinical conditions.

Technical metrics directly related to robot-mediated interaction and monitoring showed excellent test–retest stability, including end-effector recognition accuracy (ICC = 1.000) and head-orientation angle metrics (ICC = 0.958). In contrast, questionnaire-based outcomes showed moderate stability (ICC = 0.68), indicating greater variability in subjective perception across acquisitions. This lower stability is not unexpected, as usability and acceptance scores may change after repeated exposure to the robotic system, when participants become more familiar with the interaction flow and develop clearer expectations about the platform. This pattern supports the interpretation that the robotic workflow itself was reproducible, whereas participant-dependent outcomes, particularly questionnaire scores, may be influenced by familiarization, task learning, subjective perception, and day-to-day variability. Therefore, questionnaire scores were interpreted primarily as indicators of perceived usability and acceptance.

## 4 Discussion

This study presents a lightweight, safety-aware, and modular multimodal robotic pipeline for embodied HRI in post-stroke rehabilitation, with a first target application in hemispatial inattention. The proposed ROS 2-based architecture integrated therapist-mediated supervision, autonomous navigation, speech-based and graphical interaction, head-orientation monitoring, rehabilitation-oriented task execution, data logging, and usability assessment within a single structured protocol. All participants completed both acquisitions, and no adverse events or safety-relevant events requiring interruption of the experimental workflow were observed. These findings support the feasibility of implementing a supervised multimodal robotic workflow on a real mobile manipulator under controlled preclinical conditions. Consistent with this scope, the present study should be read as a technical feasibility evaluation of the robotic pipeline itself, rather than as a clinical efficacy trial or a fully powered acceptance study: its purpose is to establish that the integrated architecture can operate reliably, end-to-end, on a real mobile manipulator, which is the necessary precondition for the acceptance-oriented and patient-oriented studies described in the staged future work outlined below.

A central element of the proposed architecture is that the robot is conceived as an assistive platform embedded in a therapist-in-the-loop workflow. This design is particularly relevant in post-stroke rehabilitation, where patient status may change during the session and where fatigue, confusion, malaise, refusal, or requests for help require conservative management. Through the TCI, the therapist configures and supervises the session, while the robot autonomously manages predefined low-risk, non-contact interaction phases. Safety-relevant or recovery-related events are routed to the therapist before the rehabilitation workflow continues. This organization preserves clinical accountability while allowing the robotic system to support repetitive interaction, structured task administration, continuous monitoring, and quantitative data collection.

The results also support the use of a finite-state-machine-based dialogue architecture as the deterministic control layer of the IM. In a real mobile manipulator, dialogue management must coexist with autonomous navigation, RGB-D perception, face tracking, speech synthesis, speech recognition, graphical interaction, exercise execution, data logging, and communication with external interfaces. The proposed FSM-based architecture provides explicit state transitions, transparent safety logic, and predictable recovery strategies within these onboard computational constraints. The NLU module remains lightweight and configurable, while the FSM controls session progression, exercise confirmation, pause/resume behavior, fallback handling, and therapist escalation. This architecture represents a pragmatic compromise between conversational flexibility, hardware-aware deployment, and operational safety.

Speech-based interaction remained operational throughout the sessions, but ASR-related events were observed, especially during prolonged dialogue/questionnaire interaction. Most problematic cases involved short, fragmented, or phonetically ambiguous utterances, confirming that speech recognition is a technically vulnerable component of embodied HRI when responses are brief or acoustically similar. These findings support the use of multimodal redundancy, rule-based NLU, and FSM fallback mechanisms in safety-relevant rehabilitation interaction.

The head-orientation monitoring subsystem represents an important component of the pipeline because it links perception, interaction, and task delivery. Head orientation was used as a non-invasive operational indicator of orientation toward the robot-facing interaction area, allowing the system to generate adaptive auditory prompts when large deviations from this direction were detected. In healthy participants, attention-recall events were infrequent and head-orientation measures remained stable during verbal interaction, suggesting that RGB-D-based head-orientation monitoring can be integrated into the interaction flow without disrupting task execution.

From a clinical translation perspective, this mechanism is relevant because patients with hemispatial inattention may show reduced spontaneous orientation toward the contralesional hemispace and may require repeated external cues to maintain engagement. The proposed subsystem provides a continuous and non-invasive indicator of orientation toward the robot that can be incorporated into robot behavior. In future clinical use, this mechanism could support adaptive attention recall, help therapists quantify orientation-related behavior during exercises, and provide additional information for tailoring cueing strategies or task difficulty. The 50° threshold used in the current prototype was selected to detect large deviations of head orientation away from the robot-facing interaction area while tolerating natural head movements during speech and task execution.

The end-effector recognition task demonstrated the feasibility of synchronizing robot motion, visual observation, speech-based response acquisition, and auditory feedback. The task required participants to observe the robotic hand, interpret the verbal prompt, provide a spoken response, and receive immediate feedback within a closed-loop interaction. The high level of task completion and the limited need for repeated verbal attempts suggest that robot-mediated motor-observational tasks can be integrated into a structured rehabilitation-oriented protocol.

The screen-based visual tasks extended the evaluation beyond robot-mediated motor-observational interaction by testing whether the pipeline could also administer TCI-mediated visual exercises through the integrated TIAGo screen. Both image-recognition and shape-counting tasks were completed and logged across acquisitions, indicating that the EDM and TCI can support screen-based task delivery, item-level presentation, score computation, execution timing, and structured data recording. These tasks share components relevant to neglect-oriented assessment and rehabilitation, including visual search, target detection, sustained attention, response accuracy, completion time, and exploratory engagement. In healthy participants, differences between test and retest may reflect familiarization, greater understanding of the response format, or procedural variability.

Usability and acceptance outcomes were generally favorable. Participants perceived the platform as usable and acceptable, and repeated exposure did not negatively affect user perception. The preservation of low anxiety is particularly relevant for robotic rehabilitation, because discomfort, fear, or uncertainty toward robotic systems may reduce engagement and limit clinical adoption. These findings suggest that the proposed pipeline can provide an acceptable interaction experience in a controlled preclinical setting.

The 1-month retest showed that the complete workflow could be re-executed after repeated exposure. The same protocol was completed at both time points, with preserved task execution, comparable interaction dynamics, successful data logging, and favorable usability perception. Selected technical metrics showed high test–retest stability, supporting the operational stability of the robot-mediated interaction and monitoring components. Questionnaire-based outcomes showed greater variability, consistent with the subjective nature of usability and acceptance ratings and with possible familiarization after repeated exposure.

### 4.1 Limitations

Several limitations follow directly from the deliberate scope of this study as a technical feasibility evaluation rather than a clinical or fully powered acceptance study. First, the evaluation was conducted on 16 healthy participants, so system robustness in the presence of neglect severity, fatigue, aphasia, dysarthria, cognitive impairment, reduced compliance, and altered visuospatial exploration remains to be established in the target patient population. Second, the study did not include a formal safety-validation or failure-injection protocol: although the FSM-based architecture supports safety-aware interaction through deterministic state transitions, guard conditions, ignored out-of-sequence events, fallback counters, and therapist escalation, induced hardware or software failures were not systematically tested, and quantitative safety metrics such as emergency-stop latency, safe-state transition success rate, and recovery time were not measured.

Third, the ASR-related observations and the 1-month reliability analysis reported above are preliminary, as they were not based on a formal error-classification protocol with synchronized audio recording, independent annotators, and inter-rater agreement, nor on predefined reliability endpoints with confidence intervals and agreement limits. Finally, the current implementation should be considered a local, therapist-supervised robotic workflow: although the system generates structured digital logs and supports communication among the TCI, IM, EDM, head-orientation monitoring subsystem, and robot-side modules, integration with hospital electronic health records, remote monitoring platforms, authentication systems, interoperable clinical data-exchange standards, secure data exchange, access control, network-failure management, and cybersecurity requirements was outside the scope of this work. None of these points weaken the central claim of this study, i.e., that the integrated pipeline can be operated reliably, end-to-end, on a real mobile manipulator, but they mark the boundary between the technical feasibility demonstrated here and the acceptance- and efficacy-oriented evidence required before clinical translation, which the staged future work outlined below is designed to address.

## 5 Conclusions and future work

This study presented a safety-aware multimodal robotic pipeline for embodied HRI in post-stroke rehabilitation, implemented on the TIAGo mobile manipulator within a ROS 2-based architecture. The system integrates therapist supervision, FSM-based dialogue control, multimodal interaction, head-orientation monitoring, adaptive verbal recall, robot-mediated and screen-based task delivery, and structured data logging.

The evaluation showed that the proposed pipeline could support the complete interaction sequence across two acquisitions separated by 1 month. The system maintained synchronization among the TCI, IM, EDM, head-orientation monitoring subsystem, and robot execution layer, while supporting autonomous positioning, speech-based engagement, end-effector recognition, dialogue/questionnaire interaction, monitor-based visual tasks, usability assessment, and structured data collection. These findings support the use of the proposed architecture as an integrated framework for therapist-supervised robotic interaction in rehabilitation-oriented scenarios. The results also support the architectural choice of using an FSM-based dialogue manager as a deterministic safety backbone under real robotic hardware constraints. By combining lightweight NLU, explicit state transitions, fallback strategies, safety counters, and therapist escalation, the system allows the robot to manage predefined low-risk interaction phases while preserving therapist authority over clinically relevant decisions.

Building on the technical feasibility established in this study, future work is organized into two consecutive phases, moving from a dedicated acceptance and robustness protocol in healthy controls to a true rehabilitation protocol in post-stroke patients.

### 5.1 Phase 1: acceptance and robustness protocol in healthy controls

The next step will be a dedicated protocol, still conducted in healthy controls, specifically designed to formally test acceptance and usability of the human–robot interaction and to close the technical-validation gaps identified above. This protocol will include an adequately powered, hypothesis-driven usability and acceptance assessment; a structured safety-validation and failure-injection campaign covering ASR misclassification, loss of face tracking, ROS 2 node failure, communication interruption, invalid FSM transitions, therapist-interface failure, and stop-command activation, with quantitative measures of emergency-stop latency, safe-state transition success rate, and recovery time; and predefined test–retest reliability endpoints, including confidence intervals for ICC estimates, Bland–Altman agreement analysis, standard error of measurement, and minimal detectable change. This stage will also calibrate the head-orientation threshold against gaze-based measures and therapist observation.

### 5.2 Phase 2: rehabilitation protocol in post-stroke patients

Once acceptance and technical robustness are formally established, the pipeline will be evaluated in a true rehabilitation protocol involving post-stroke patients with hemispatial inattention. This stage will use an adequately powered patient sample and predefined clinical endpoints to assess rehabilitation outcomes, functional recovery, transfer to activities of daily living, long-term adherence, and responsiveness to treatment, while evaluating system robustness in the presence of neglect severity, fatigue, aphasia, dysarthria, cognitive impairment, reduced compliance, and altered visuospatial exploration. Screen-based and head-orientation measures will be related to standardized neglect scales to support their clinical interpretation, and additional rehabilitation exercises, including arm-pointing and mirroring tasks, will be integrated. The adaptive task-progression component of the pipeline will also be extended through more refined difficulty adaptation, structured progression rules, and therapist-facing performance summaries, with clinical decisions regarding task progression, interruption, simplification, or termination remaining under therapist supervision. Extending the system toward a connected digital health infrastructure is currently beyond the scope of our research. Overall, the proposed system represents a translational step toward robotic platforms that can support interaction, head-orientation monitoring, quantitative assessment, and adaptive task delivery within therapist-supervised neurorehabilitation workflows.

## Data Availability

The raw data supporting the conclusions of this article will be made available by the authors, without undue reservation.
