# Supplementary material for: Preclinical evaluation of a safety-aware multimodal robotic pipeline for human–robot interaction in post-stroke rehabilitation
Source: Front Digit Health. 2026 Oct 9;8:1951309. doi: 10.3389/fdgth.2026.1951309 (PMC13690936; doi:10.3389/fdgth.2026.1951309)
Supplement: Supplementary file 1 [file Table1.docx]

Supplementary Material

# 1 Supplementary Tables

Table S1. Canonical intent set and NLU matching policy

| **Input source** | **Canonical intent / event** | **Representative inputs** | **Matching priority** | **Acceptance criterion** | **Default FSM handling** |
| --- | --- | --- | --- | --- | --- |
| TCI / system | START_SESSION | Therapist starts the session from the TCI | Direct event mapping | Valid TCI event | Loads participant/session data, generates greeting and start-confirmation prompt. |
| TCI / EDM | INTRODUCE_EXERCISE | Exercise introduction event from TCI/EDM | Direct event mapping | Valid event in an admissible FSM state | Loads the exercise, presents the task, and asks for exercise confirmation. |
| TCI / EDM | BEGIN_EXERCISE | Direct exercise-start event from TCI/EDM | Direct event mapping | Valid event in an admissible FSM state | Loads the exercise and enters exercise execution without additional patient confirmation. |
| TCI / EDM | EXERCISE_RESULT | Exercise completion message with score/result payload | Direct event mapping | Accepted only if the FSM is in EXERCISE_RUNNING | Evaluates the exercise result, emits structured feedback/event, and returns to exercise-selection/progression state. |
| TCI / system | END_SESSION | Therapist/session-closure command | Direct event mapping | Valid end-session event | Closes the session with the stored session outcome. Duplicate closure events are ignored and logged. |
| Therapist interface | RESUME_SESSION | Therapist selects resume | Direct therapist decision | Accepted only in WAITING_THERAPIST_DECISION | Resumes the supervised workflow. |
| Therapist interface | REPEAT_EXERCISE | Therapist selects repeat exercise | Direct therapist decision | Accepted only in WAITING_THERAPIST_DECISION | Reintroduces the current exercise and asks for confirmation. |
| Therapist interface | SKIP_EXERCISE | Therapist selects skip exercise | Direct therapist decision | Accepted only in WAITING_THERAPIST_DECISION | Skips the current exercise and waits for the next exercise. |
| Therapist interface | END_SESSION | Therapist selects end session | Direct therapist decision | Accepted in WAITING_THERAPIST_DECISION | Ends the session with the stored outcome. |
| Speech | CONFIRM | “sì”, “si”, “ok”, “va bene”, “iniziamo”, “proviamo”, “sono pronta”, “sono pronto” | Exact match; keyword/rule match; similarity recovery | Exact canonical match or similarity score ≥ T_sim after normalization | Advances the pending confirmation state when confirmation is expected. |
| Speech | REFUSE | “no”, “non voglio”, “non mi va”, “non sono pronta”, “non sono pronto”, “non me la sento” | Exact match; negation/rule-based matching; similarity recovery | Presence of refusal/negation cue or similarity score ≥ T_sim | First refusal triggers reassurance and reprompt; repeated refusal triggers therapist escalation. |
| Speech | REQUEST_PAUSE | “pausa”, “facciamo una pausa”, “sono stanca”, “sono stanco”, “ho bisogno di una pausa” | Exact match; keyword/rule-based matching | Pause/fatigue cue detected | During exercise, moves to PAUSED; before starting, treated as temporary non-readiness and managed by reprompt/escalation policy. |
| Speech | REQUEST_STOP | “basta”, “fermiamoci”, “voglio fermarmi”, “non ce la faccio più”, “non voglio continuare” | Exact match; keyword/rule-based matching | Stop/interruption cue detected | Suspends or interrupts autonomous progression and triggers therapist supervision depending on current state. |
| Speech | REQUEST_HELP | “aiuto”, “mi aiuti?”, “ho bisogno di aiuto”, “non riesco” | Exact match; keyword/rule-based matching | Help cue detected | Safety-relevant global intent; immediately triggers therapist alert. |
| Speech | MALAISE | “sto male”, “ho male”, “mi gira la testa”, “non mi sento bene”, “male” | Exact match; keyword/rule-based matching | Malaise/discomfort cue detected | Safety-relevant global intent; stops autonomous progression and triggers therapist alert. |
| Speech | CONFUSION | “non ho capito”, “sono confusa”, “sono confuso”, “non capisco” | Exact match; keyword/rule-based matching | Confusion cue detected | Produces clarification or repetition; repeated unresolved confusion may trigger therapist escalation. |
| Speech | ASK_REPEAT | “puoi ripetere?”, “ripeti”, “non ho sentito”, “cosa hai detto?” | Exact match; keyword/rule-based matching | Repetition cue detected | Repeats the pending instruction without changing the current FSM state. |
| Speech | ASK_ROBOT_IDENTITY | “chi sei?”, “sei un robot?”, “come ti chiami?” | Exact match; keyword/rule-based matching | Robot-identity question detected | State-preserving answer followed by contextual reprompt. |
| Speech | ASK_SESSION_EXPLANATION | “cosa facciamo oggi?”, “che cosa facciamo oggi?”, “mi spieghi la sessione?” | Exact match; keyword/rule-based matching | Session-explanation question detected | State-preserving answer followed by contextual reprompt. |
| Speech | ASK_CURRENT_TASK | “che si fa?”, “cosa devo fare?”, “cosa stiamo facendo?” | Exact match; keyword/rule-based matching | Current-task question detected | State-preserving answer followed by contextual reprompt. |
| Speech | ASK_EXERCISE_REASON | “perché dobbiamo farlo?”, “a cosa serve?”, “perché facciamo questo esercizio?” | Keyword/rule-based matching | Exercise-reason question detected | State-preserving answer followed by contextual reprompt. |
| Speech | ASK_REMAINING_TIME | “quanto manca?”, “quanto dura?”, “quanto tempo resta?” | Keyword/rule-based matching | Time/duration question detected | State-preserving answer followed by contextual reprompt. |
| Speech | ASK_CAN_PAUSE | “posso fare una pausa?”, “posso fermarmi un attimo?” | Keyword/rule-based matching | Permission-to-pause question detected | Provides information about available control options without automatically pausing. |
| Speech | ASK_CAN_STOP | “posso smettere?”, “posso interrompere?”, “posso fermarmi?” | Keyword/rule-based matching | Permission-to-stop question detected | Provides information about available control options without automatically ending the session. |
| Speech | UNKNOWN | Ambiguous, incomplete, non-matching, or low-reliability utterances | Fallback class | No exact/rule match and similarity score < T_sim | Outside task execution, increments fallback counter and may trigger clarification or escalation. During EXERCISE_RUNNING, task-specific answers may remain ignored by the dialogue FSM if no safety intent is detected. |
| Speech | OUT_OF_CONTEXT | Utterances unrelated to the active rehabilitation interaction | Fallback/rule-based class | Classified as unrelated to current task/session context | State-preserving fallback; repeated events may trigger therapist escalation. |

**Notes.** Text normalization was applied before speech matching and included lowercasing, accent normalization, punctuation removal, whitespace normalization, and minor orthographic normalization. Safety-relevant intents, including MALAISE, REQUEST_HELP, and REQUEST_STOP, were assigned priority over task-related and generic fallback handling. The similarity score was used only as an internal rule-based acceptance criterion and was not interpreted as a calibrated probabilistic confidence value.

Table S2. FSM state-transition matrix

| **Current FSM state** | **Input / event** | **Condition / counter policy** | **FSM output / logged event** | **Next FSM state** |
| --- | --- | --- | --- | --- |
| WAITING_SESSION_START | START_SESSION | Valid start command from TCI | Robot greeting, session explanation, start-confirmation prompt; STATE_CHANGED logged | WAITING_START_CONFIRMATION |
| WAITING_SESSION_START | EXERCISE_RESULT | Result received before session/exercise is active | EXERCISE_RESULT_IGNORED; event logged as out-of-sequence | WAITING_SESSION_START |
| WAITING_SESSION_START | INTRODUCE_EXERCISE or BEGIN_EXERCISE | Exercise event received before session confirmation | EXERCISE_INTRODUCTION_IGNORED or EXERCISE_BEGIN_IGNORED; event logged | WAITING_SESSION_START |
| WAITING_SESSION_START | END_SESSION | Session already closed or not active | SESSION_END_IGNORED; duplicate/invalid closure logged | WAITING_SESSION_START |
| WAITING_START_CONFIRMATION | CONFIRM | Patient confirms session start | PATIENT_CONFIRMED_SESSION_START; STATUS_SUCCESS for start confirmation | WAITING_EXERCISE_START |
| WAITING_START_CONFIRMATION | First REFUSE | session_refusal_count = 1 | Reassurance and reprompt; PATIENT_REFUSED_SESSION_START logged | WAITING_START_CONFIRMATION |
| WAITING_START_CONFIRMATION | Repeated REFUSE | session_refusal_count ≥ 2 | STATUS_REFUSED; therapist alert for repeated session refusal | WAITING_THERAPIST_DECISION |
| WAITING_START_CONFIRMATION | REQUEST_PAUSE / fatigue before start | First occurrence treated as temporary non-readiness | Reassurance and start reprompt; event logged | WAITING_START_CONFIRMATION |
| WAITING_START_CONFIRMATION | Repeated REQUEST_PAUSE / persistent non-readiness | Repeated non-readiness according to counter policy | Therapist alert; autonomous start is suspended | WAITING_THERAPIST_DECISION |
| WAITING_START_CONFIRMATION | MALAISE | Immediate safety escalation | PATIENT_REPORTED_MALAISE; therapist alert; session outcome set to malaise/failure | WAITING_THERAPIST_DECISION |
| WAITING_START_CONFIRMATION | REQUEST_HELP | Immediate safety escalation | PATIENT_REQUESTED_HELP; therapist alert | WAITING_THERAPIST_DECISION |
| WAITING_START_CONFIRMATION | REQUEST_STOP | Patient requests interruption before session start | Stop/interruption event logged; therapist supervision requested | WAITING_THERAPIST_DECISION |
| WAITING_START_CONFIRMATION | Lateral informational question | Robot/session/task question while confirmation is pending | State-preserving answer and contextual reprompt | WAITING_START_CONFIRMATION |
| WAITING_START_CONFIRMATION | ASK_REPEAT | Patient asks to repeat | Repeats pending start-confirmation prompt | WAITING_START_CONFIRMATION |
| WAITING_START_CONFIRMATION | UNKNOWN or OUT_OF_CONTEXT | Counter below escalation limit | Clarification/unknown response; counter incremented | WAITING_START_CONFIRMATION |
| WAITING_START_CONFIRMATION | Repeated UNKNOWN or OUT_OF_CONTEXT | Counter reaches escalation limit, e.g., three unresolved fallback events | Therapist alert for interaction breakdown / possible ASR or comprehension failure | WAITING_THERAPIST_DECISION |
| WAITING_EXERCISE_START | INTRODUCE_EXERCISE | Valid exercise-introduction event | Exercise loaded; robot introduces task and asks for exercise confirmation | WAITING_EXERCISE_CONFIRMATION |
| WAITING_EXERCISE_START | BEGIN_EXERCISE | Valid direct exercise-start event | Exercise loaded and started without additional confirmation | EXERCISE_RUNNING |
| WAITING_EXERCISE_START | END_SESSION | Valid session closure after exercise sequence or operator decision | Outcome-aware session closure; final status logged | WAITING_SESSION_START |
| WAITING_EXERCISE_START | EXERCISE_RESULT | Result received when no exercise is running | EXERCISE_RESULT_IGNORED; event logged | WAITING_EXERCISE_START |
| WAITING_EXERCISE_START | REQUEST_HELP, MALAISE, REQUEST_STOP | Safety-relevant input before next exercise | Therapist alert; autonomous progression suspended | WAITING_THERAPIST_DECISION |
| WAITING_EXERCISE_START | Lateral informational question | Patient asks about current task/session | State-preserving answer and contextual reprompt | WAITING_EXERCISE_START |
| WAITING_EXERCISE_CONFIRMATION | CONFIRM | Patient confirms exercise start | PATIENT_CONFIRMED_EXERCISE; exercise begins | EXERCISE_RUNNING |
| WAITING_EXERCISE_CONFIRMATION | First REFUSE | exercise_refusal_count = 1 | Reassurance and exercise-confirmation reprompt | WAITING_EXERCISE_CONFIRMATION |
| WAITING_EXERCISE_CONFIRMATION | Repeated REFUSE | exercise_refusal_count ≥ 2 | Therapist alert for repeated exercise refusal | WAITING_THERAPIST_DECISION |
| WAITING_EXERCISE_CONFIRMATION | REQUEST_PAUSE / non-readiness | First occurrence | Reassurance and exercise-confirmation reprompt | WAITING_EXERCISE_CONFIRMATION |
| WAITING_EXERCISE_CONFIRMATION | MALAISE | Immediate safety escalation | PATIENT_REPORTED_MALAISE; therapist alert | WAITING_THERAPIST_DECISION |
| WAITING_EXERCISE_CONFIRMATION | REQUEST_HELP | Immediate safety escalation | PATIENT_REQUESTED_HELP; therapist alert | WAITING_THERAPIST_DECISION |
| WAITING_EXERCISE_CONFIRMATION | REQUEST_STOP | Patient requests interruption before exercise | Stop/interruption event logged; therapist alert | WAITING_THERAPIST_DECISION |
| WAITING_EXERCISE_CONFIRMATION | Lateral informational question | Patient asks about task, reason, robot, or time | State-preserving answer and exercise-confirmation reprompt | WAITING_EXERCISE_CONFIRMATION |
| WAITING_EXERCISE_CONFIRMATION | EXERCISE_RESULT | Result received before exercise start | EXERCISE_RESULT_IGNORED; event logged | WAITING_EXERCISE_CONFIRMATION |
| EXERCISE_RUNNING | Task-specific spoken answer not mapped to safety intent | Examples: numbers, object names, uncertain task answers | No robot dialogue output; answer is not treated as a dialogue command; state preserved | EXERCISE_RUNNING |
| EXERCISE_RUNNING | REQUEST_PAUSE | Patient asks to pause | Pause confirmed; current exercise context preserved | PAUSED |
| EXERCISE_RUNNING | REQUEST_STOP | Patient requests interruption during exercise | Exercise/session progression suspended; therapist supervision requested according to policy | WAITING_THERAPIST_DECISION |
| EXERCISE_RUNNING | REQUEST_HELP | Immediate safety escalation | PATIENT_REQUESTED_HELP; therapist alert | WAITING_THERAPIST_DECISION |
| EXERCISE_RUNNING | MALAISE | Immediate safety escalation | PATIENT_REPORTED_MALAISE; therapist alert; autonomous progression stopped | WAITING_THERAPIST_DECISION |
| EXERCISE_RUNNING | REFUSE | Patient refuses during exercise | Refusal event logged; therapist supervision requested | WAITING_THERAPIST_DECISION |
| EXERCISE_RUNNING | Lateral informational question | Patient asks question during exercise | State-preserving answer if allowed by policy; exercise state maintained | EXERCISE_RUNNING |
| EXERCISE_RUNNING | EXERCISE_RESULT | Valid exercise-completion result received | Score evaluated; structured result, outcome, and recommendation emitted | WAITING_EXERCISE_START |
| EXERCISE_RUNNING | Invalid exercise/session event | Event not admissible in running state | Ignored-event log generated; state preserved | EXERCISE_RUNNING |
| PAUSED | CONFIRM / resume confirmation | Patient agrees to resume | Resume utterance/event; current exercise context restored | EXERCISE_RUNNING |
| PAUSED | REQUEST_PAUSE | Pause already active | Pause-state reminder; event logged | PAUSED |
| PAUSED | ASK_REPEAT or unclear input | Patient asks for repetition or remains uncertain | Pause instruction repeated | PAUSED |
| PAUSED | REQUEST_STOP or REFUSE | Patient does not want to continue | Therapist alert for stop/interruption during pause | WAITING_THERAPIST_DECISION |
| PAUSED | MALAISE | Immediate safety escalation | PATIENT_REPORTED_MALAISE; therapist alert | WAITING_THERAPIST_DECISION |
| PAUSED | REQUEST_HELP | Immediate safety escalation | PATIENT_REQUESTED_HELP; therapist alert | WAITING_THERAPIST_DECISION |
| WAITING_THERAPIST_DECISION | RESUME_SESSION | Valid therapist decision | Therapist decision logged; workflow resumes | WAITING_EXERCISE_START |
| WAITING_THERAPIST_DECISION | REPEAT_EXERCISE | Valid therapist decision | Current exercise reintroduced; exercise-confirmation prompt generated | WAITING_EXERCISE_CONFIRMATION |
| WAITING_THERAPIST_DECISION | SKIP_EXERCISE | Valid therapist decision | Current exercise skipped; system waits for next exercise | WAITING_EXERCISE_START |
| WAITING_THERAPIST_DECISION | END_SESSION | Valid therapist decision or TCI closure | Session closed with stored outcome | WAITING_SESSION_START |
| WAITING_THERAPIST_DECISION | BEGIN_EXERCISE, INTRODUCE_EXERCISE, or EXERCISE_RESULT | Event received before therapist decision | Event ignored and logged as out-of-sequence | WAITING_THERAPIST_DECISION |
| WAITING_THERAPIST_DECISION | Unrecognized therapist decision | Decision not mapped to allowed options | Therapist-decision unknown event logged; system remains suspended | WAITING_THERAPIST_DECISION |
| CLOSING / ERROR | Reset or closure event | Manual/system recovery condition | Session reset or safe closure logged | WAITING_SESSION_START |

Table S3. I-UTAUT questionnaire items used in the acceptance assessment

| **Construct / domain** | **Item code** | **Italian item wording** | **English translation** | **Scoring direction** |
| --- | --- | --- | --- | --- |
| Computer/Internet familiarity | PRE1 | Come descriveresti la tua conoscenza generale dei computer? | How would you describe your general knowledge of computers? | Higher = greater familiarity |
| Computer/Internet familiarity | PRE2 | Come descriveresti la tua conoscenza di Internet? | How would you describe your knowledge of the Internet? | Higher = greater familiarity |
| Computer/Internet familiarity | PRE3 | Quanto spesso usi Internet ogni giorno? | How often do you use the Internet every day? | Higher = greater use/familiarity |
| Anxiety | ANX1 | Se dovessi usare TIAGo, avrei paura di fare degli errori | If I had to use TIAGo, I would be afraid of making mistakes | Higher = greater anxiety |
| Anxiety | ANX2 | Se dovessi usare TIAGo, avrei paura di rompere qualcosa | If I had to use TIAGo, I would be afraid of breaking something | Higher = greater anxiety |
| Anxiety | ANX3 | Trovo che TIAGo mi faccia paura | I find TIAGo frightening | Higher = greater anxiety |
| Anxiety | ANX4 | Trovo che TIAGo sia minaccioso | I find TIAGo threatening | Higher = greater anxiety |
| Attitude toward use | ATT1 | Penso che sia una buona idea usare TIAGo | I think using TIAGo is a good idea | Higher = more positive acceptance |
| Attitude toward use | ATT2 | TIAGo potrebbe rendere la mia vita più interessante | TIAGo could make my life more interesting | Higher = more positive acceptance |
| Attitude toward use | ATT3 | È una buona idea usare TIAGo | It is a good idea to use TIAGo | Higher = more positive acceptance |
| Facilitating conditions / resources | FC1 | Ho tutto ciò di cui ho bisogno per poter utilizzare TIAGo | I have everything I need to use TIAGo | Higher = more positive acceptance |
| Knowledge / perceived ability | KNOW1 | So abbastanza di TIAGo per farne buon uso | I know enough about TIAGo to make good use of it | Higher = more positive acceptance |
| Intention to use | INT1 | Penso che userò TIAGo nei prossimi giorni | I think I will use TIAGo in the next few days | Higher = greater intention to use |
| Intention to use | INT2 | Sono certo di usare TIAGo nei prossimi giorni | I am sure I will use TIAGo in the next few days | Higher = greater intention to use |
| Intention to use | INT3 | Sto pianificando di usare TIAGo nei prossimi giorni | I am planning to use TIAGo in the next few days | Higher = greater intention to use |
| Adaptability | AD1 | Penso che TIAGo sia capace di adattarsi ai miei bisogni | I think TIAGo is able to adapt to my needs | Higher = more positive acceptance |
| Adaptability | AD2 | Penso che TIAGo farà solo ciò di cui ho bisogno in quel preciso momento | I think TIAGo will do only what I need at that specific moment | Higher = more positive acceptance |
| Adaptability | AD3 | Penso che TIAGo mi aiuterà quando io lo riterrò necessario | I think TIAGo will help me when I consider it necessary | Higher = more positive acceptance |
| Enjoyment / pleasantness | ENJ1 | Trovo piacevole che TIAGo mi parli | I find it pleasant that TIAGo talks to me | Higher = more positive acceptance |
| Enjoyment / pleasantness | ENJ2 | Mi piace svolgere attività con TIAGo | I like doing activities with TIAGo | Higher = more positive acceptance |
| Enjoyment / pleasantness | ENJ3 | Penso che TIAGo sia piacevole | I think TIAGo is pleasant | Higher = more positive acceptance |
| Enjoyment / interest | ENJ4 | Penso che TIAGo sia interessante | I think TIAGo is interesting | Higher = more positive acceptance |
| Enjoyment / boredom | ENJ5 | Penso che TIAGo sia noioso | I think TIAGo is boring | Reverse-coded; higher raw score = more negative perception |
| Ease of use | EOU1 | Credo che imparerò presto come usare TIAGo | I believe I will quickly learn how to use TIAGo | Higher = greater perceived ease of use |
| Ease of use | EOU2 | Trovo che TIAGo sia semplice da usare | I find TIAGo easy to use | Higher = greater perceived ease of use |
| Self-efficacy | SE1 | Penso di poter utilizzare TIAGo senza alcun aiuto | I think I can use TIAGo without any help | Higher = greater perceived self-efficacy |
| Facilitating conditions / assistance | FC2 | Penso di poter usare TIAGo quando c’è qualcuno che può aiutarmi | I think I can use TIAGo when there is someone who can help me | Higher = more positive perceived support |
| Facilitating conditions / manual | FC3 | Penso di poter usare TIAGo grazie ad un buon manuale | I think I can use TIAGo with the help of a good manual | Higher = more positive perceived support |
| Sociability | SOC1 | Ritengo che TIAGo sia un buon compagno per fare una conversazione | I consider TIAGo a good companion for a conversation | Higher = greater perceived sociability |
| Sociability / interaction pleasantness | SOC2 | Trovo sia piacevole interagire con TIAGo | I find it pleasant to interact with TIAGo | Higher = greater perceived sociability |
| Understanding / social interaction | SOC3 | Sento che TIAGo mi capisce | I feel that TIAGo understands me | Higher = greater perceived sociability/understanding |
| Sociability / pleasantness | SOC4 | Credo che TIAGo sia gradevole | I believe TIAGo is pleasant | Higher = greater perceived sociability |
| Perceived usefulness | PU1 | Credo che TIAGo mi sia utile | I believe TIAGo is useful to me | Higher = greater perceived usefulness |
| Perceived usefulness | PU2 | Sarebbe utile per me avere TIAGo | It would be useful for me to have TIAGo | Higher = greater perceived usefulness |
| Perceived usefulness | PU3 | Penso che TIAGo possa aiutarmi in molte cose | I think TIAGo can help me with many things | Higher = greater perceived usefulness |
| Social influence | SI1 | Penso che i miei familiari e le persone che mi aiutano apprezzerebbero se usassi TIAGo | I think my family members and the people who help me would appreciate it if I used TIAGo | Higher = greater perceived social influence |
| Social influence | SI2 | Penso che darei una buona impressione se usassi TIAGo | I think I would make a good impression if I used TIAGo | Higher = greater perceived social influence |
| Social presence / anthropomorphism | SP1 | Interagendo con TIAGo, mi è sembrato di parlare con una persona vera | While interacting with TIAGo, it seemed to me that I was talking to a real person | Higher = greater perceived social presence |
| Social presence / gaze perception | SP2 | Talvolta mi è sembrato che TIAGo mi guardasse davvero | Sometimes it seemed to me that TIAGo was really looking at me | Higher = greater perceived social presence |
| Anthropomorphism | SP3 | Riesco ad immaginare TIAGo come una creatura vivente | I can imagine TIAGo as a living creature | Higher = greater anthropomorphic perception |
| Reverse social presence | SP4 | Penso spesso che TIAGo non sia una persona vera | I often think that TIAGo is not a real person | Reverse-coded depending on social-presence scoring |
| Social presence / emotional attribution | SP5 | Talvolta sembra che TIAGo abbia sentimenti veri | Sometimes it seems that TIAGo has real feelings | Higher = greater anthropomorphic perception |
| Trust | TR1 | Mi fiderei di TIAGo se mi desse dei consigli | I would trust TIAGo if it gave me advice | Higher = greater trust |
| Trust / advice adherence | TR2 | Seguirei i consigli suggeriti da TIAGo | I would follow the advice suggested by TIAGo | Higher = greater trust |
